# A Weavable and Scalable Cotton‐Yarn‐Based Battery Activated by Human Sweat for Textile Electronics

**DOI:** 10.1002/advs.202103822

**Published:** 2022-01-06

**Authors:** Gang Xiao, Jun Ju, Hao Lu, Xuemei Shi, Xin Wang, Wei Wang, Qingyou Xia, Guangdong Zhou, Wei Sun, Chang Ming Li, Yan Qiao, Zhisong Lu

**Affiliations:** ^1^ Institute for Clean Energy & Advanced Materials School of Materials & Energy Southwest University Chongqing 400715 P. R. China; ^2^ College of Food Science Southwest University Chongqing 400715 P. R. China; ^3^ Singapore Institute of Manufacturing Technology Singapore 138669 Singapore; ^4^ Biological Science Research Center Academy for Advanced Interdisciplinary Studies Southwest University Chongqing 400715 P. R. China; ^5^ College of Artificial Intelligence Chongqing Key Laboratory of Brain‐inspired Computing & Intelligent Control Southwest University Chongqing 400715 P. R. China; ^6^ Key Laboratory of Laser Technology and Optoelectronic Functional Materials of Hainan Province College of Chemistry and Chemical Engineering Hainan Normal University Haikou 571158 P. R. China; ^7^ School of Materials Science and Engineering Suzhou University of Science and Technology Suzhou 215011 P. R. China

**Keywords:** biocompatibility, cotton‐yarn‐based batteries, sweat‐activated battery, textile electronics, weavability

## Abstract

Sweat‐activated batteries (SABs) are lightweight, biocompatible energy generators that produce sufficient power for skin‐interface electronic devices. However, the fabrication of 1D SABs that are compatible with conventional textile techniques for self‐powered wearable electronics remains challenging. In this study, a cotton‐yarn‐based SAB (CYSAB) with a segmental structure is developed, in which carbon‐black‐modified, pristine yarn and Zn foil‐wrapped segments are prepared to serve as the cathode, salt bridge, and anode, respectively. Upon electrolyte absorption, the CYSAB can be rapidly activated. Its performance is closely related to the ion concentration, infiltrated electrolyte volume, and evaporation rate. The CYSAB can tolerate repeated bending and washing without any significant influence on its power output. Moreover, the CYSABs can be woven into fabrics and connected in series and parallel configurations to produce an energy supplying headband, which can be activated by the sweat secreted from a volunteer during a cycling exercise to power light‐emitting diode headlights. The developed CYSAB can also be integrated with yarn‐based strain sensors to achieve a smart textile for the self‐powered sensing of human motion and breathing. This weavable, washable, and scalable CYSAB is expected to contribute to the manufacturing of self‐powered smart textiles for future applications in wearable healthcare monitoring.

## Graphical abstract

Based on the spontaneous liquid absorption and transport properties of cotton yarn, a 1D sweat‐activated battery (SAB) with great flexibility, washability, and reusability is fabricated. This cotton‐yarn‐based SAB can be perfectly integrated into fabrics via conventional textile techniques in both series and parallel configurations to function as a biocompatible and reliable power source for self‐powered human exercise sensing applications.

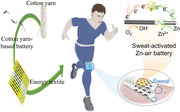

## 1 Introduction

Wearable electronics are involved in many aspects of cutting‐edge research in the fields of sensors, healthcare, electronic displays, smart textiles, and Internet of Things (IoT).^[^ ^1^, ^2^, ^3^ ^]^ The past 20 years have witnessed the rapid development of wearable electronics and the emergence of diverse wearable devices, such as flexible sensors/actuators, optoelectronic devices, electronic display systems, field‐effect transistors, electronic textiles, and soft electronic skin.^[^ ^3^, ^4^, ^5^, ^6^, ^7^, ^8^, ^9^ ^]^ An electrical power source is usually required to maintain the long‐term operation of these devices. Currently, commercialized coin cells and miniaturized lithium‐ion batteries are still the most widely used power sources, but their size, thickness, weight, and rigidity cannot meet the requirements of wearable systems including skin‐conformability, flexibility/stretchability, and portability.^[^ ^2^, ^10^, ^11^, ^12^ ^]^ Recently, flexible batteries with sandwiched structures and core‐sheath designs have been developed to provide a consistent energy supply to wearable systems, but the common use of alkaline electrolytes greatly hinders their application in skin‐interfaced wearable electronics.^[^ ^13^, ^14^, ^15^, ^16^ ^]^

Sweat can be easily collected from the human body in a noninvasive manner, and it contains a variety of substances including ions, peptides, metabolites, and small molecules.^[^ ^17^ ^]^ Thin‐film‐ or textile‐based enzymatic biofuel cells have been developed to efficiently convert the chemical energy stored in these sweat constituents into electricity.^[^ ^18^, ^19^, ^20^ ^]^ Although flexible biofuel cells may have the potential to power wearable electronic devices, the most significant challenge associated with these cells is their unstable output, which is dependent on the concentrations of lactic acid and glucose in sweat. Because the power produced by biofuel cells cannot stably support the long‐term operation of electronic devices, an additional energy storage unit is required. In addition to biofuel molecules, positive and negative ions (i.e., Na^+^, K^+^, Cl^−^) in sweat render it a biocompatible, environmentally friendly, and reliable electrolyte for wearable energy devices.^[^ ^21^ ^]^ By combining the advantages of sweat as an electrolyte and metal batteries, the development of sweat‐activated batteries (SABs) has been realized over the past few years. A SAB consisting of Mg anode, filter paper, and Ag/AgCl cathode was previously developed to monitor the conductivity of sweat.^[^ ^22^ ^]^ Furthermore, a biocompatible Mg‐Ag/AgCl SAB embedded within a paper‐based microfluidic platform was constructed to simultaneously power on‐skin devices for monitoring the heart rate, sweat chloride, and sweat pH of participants.^[^ ^23^ ^]^ A Zn film and a carbon‐nanotube‐coated Cu film were deposited on paper to produce an interdigitated structure and serve as the anode and cathode, respectively.^[^ ^24^ ^]^ Upon sweat absorption, the battery could be switched on to power the sensing of the heart rate and lactic acid levels of a participant. A Zn foil, paper, and free‐standing polyaniline/carbon nanotube film was also developed to form a sweat‐chargeable on‐skin supercapacitor, in which sweat was absorbed by the paper to activate a Zn‐air battery for supercapacitor charging.^[^ ^25^ ^]^ These examples demonstrate that paper has been widely used as both a sweat absorption layer and an electrode spacer in current SABs owing to its low cost, high availability, porous microstructure, and strong water absorption capability.

An ideal wearable energy supply system should adapt to human motion and maintain its electrochemical properties during the wearing process. Paper‐based SABs can tolerate repeated bending/twisting, but the poor tensile strength of paper greatly hinders their practical applications. Very recently, a sweat‐activated Zn‐Ag_2_O battery was prepared on textiles using the screen‐printing technique.^[^ ^26^ ^]^ Textile‐supported SABs exhibit great stretchability, but their fabrication is highly dependent on the ink quality. The washability of screen‐printed electrodes is another concern for the repeated use of textile‐based SABs. In comparison to 2D devices based on paper and textiles, 1D SABs should be more attractive owing to their excellent compatibility with textile electronics and implantable medical devices.^[^ ^27^, ^28^, ^29^, ^30^ ^]^ However, to the best of our knowledge, no studies have been conducted concerning wearable, weavable, and scalable 1D SABs.

Yarn has been regarded as an ideal support for wearable electronic devices because of its light weight, low cost, excellent flexibility, and ease of weaving/knitting/stitching.^[^ ^31^, ^32^ ^]^ The sweat wicking capabilities of various yarns have been investigated for the design of fast‐dry clothes.^[^ ^33^, ^34^ ^]^ In our previous studies, yarn‐based microfluidic devices were prepared to collect sweat samples from the human body for the on‐body measurement of sweat glucose, lactate, and pH levels.^[^ ^35^, ^36^ ^]^ Yarn is a long continuous strand of fibers with interlocked structures, in which micro/nanosized channels between neighboring fibers enable the spontaneous transport of liquid via capillary forces.^[^ ^37^, ^38^, ^39^ ^]^ Therefore, yarn may be used as a favorable sweat absorption substrate to fabricate a 1D SAB, which could be further integrated into fabrics to sustainably power wearable electronic systems. In this study, we developed a new type of flexible, weavable, and scalable cotton‐yarn‐based sweat‐activated battery (CYSAB) with a segmented structure consisting of carbon‐black‐modified, salt bridge, and Zn‐foil‐wrapped segments. The carbon black segment serves as the cathode to catalyze the oxygen reduction reaction. The Zn‐foil‐wrapped component functions as an anode to provide a chemical energy source. The sweat electrolyte can be rapidly wicked by the yarn and transported between the two electrodes along the bridge to facilitate the redox reaction. Upon sweat wicking, the CYSAB can be activated to produce energy. A number of CYSABs can be connected in series or parallel to form a battery pack to provide sufficient power. Moreover, multiple CYSABs could be fabricated into a long yarn segment, enabling the scale‐up of CYSAB production and their integration with the textile industry. Energy supplying fabrics woven using CYSABs may be designed as headbands and chestbands to harvest sweat from volunteers during exercise, further providing energy to drive LEDs as indicators and strain sensors for motion monitoring. The as‐prepared CYSABs may enhance the functionality of textiles for diverse wearable applications.

## 2 Results and Discussion

**Figure 1:**
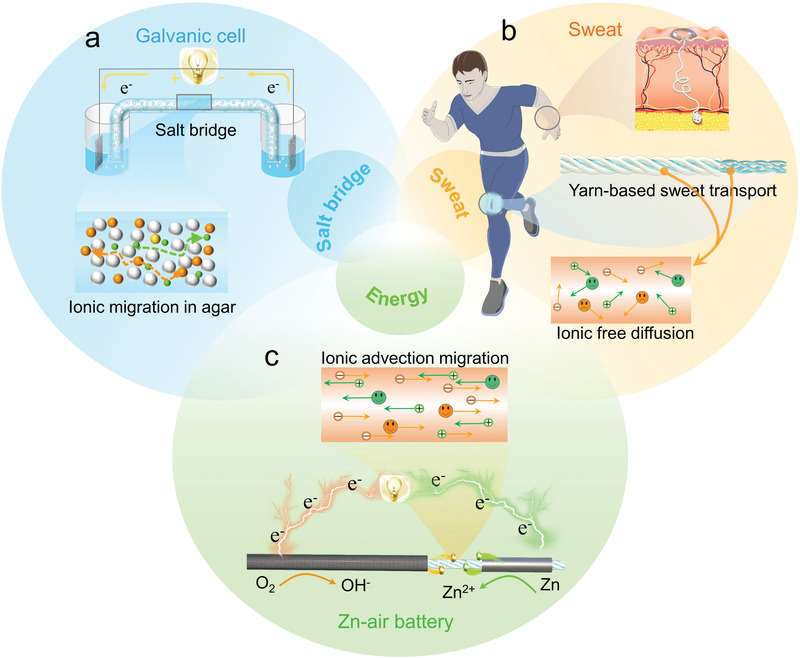
a) Structure and working principle of a salt bridge. b) Yarn‐based sweat reservoir and transport channels. c) Design of the cotton‐yarn‐based sweat‐activated battery.

A salt bridge is a connection between the oxidation and reduction half‐cells of a galvanic cell that serves to maintain electrical neutrality within the internal circuit. Without a salt bridge, negative or positive charges quickly accumulate in the respective half‐cells, preventing further reactions and the production of electricity. A salt bridge is usually made of a porous material filled with an electrolyte, in which the positive and negative ions move freely toward the cathode and anode, respectively, to balance the charges in the two compartments (**Figure** **1**a). Sweat is a transparent liquid secreted by the eccrine glands and contains a wealth of chemicals such as ions, metabolites, hormones, small proteins, and peptides.^[^ ^40^ ^]^ Among these, Na^+^, Cl^−^, K^+^, and Ca^2+^ are the most abundant components, which endow sweat with natural, safe, and reliable electrolyte properties. When sweat is wicked by a cotton yarn via capillary force, gaps between the fibers act as reservoirs and transport channels for sweat,^[^ ^35^, ^39^ ^]^ allowing the free navigation of ions along the channels (Figure 1b). Therefore, the sweat‐infiltrated yarn may serve as a salt bridge to connect the anode and cathode in a metal battery. Inspired by the configuration of a conventional galvanic cell, we designed a cotton‐yarn‐based sweat‐activated battery with a three‐segment structure, in which carbon‐black‐modified anterior, pristine middle, and Zn‐foil‐wrapped posterior segments act as the cathode, salt bridge, and anode, respectively (Figure 1c). In the presence of perspiration, sweat absorbed by the yarn may activate the CYSAB, thereby producing energy to power electronic devices.

**Figure 2:**
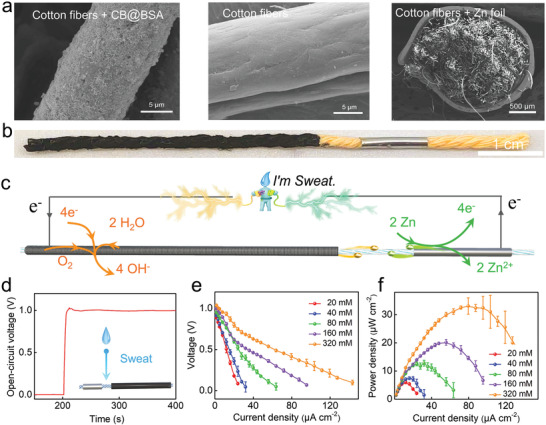
a) Morphologies of the carbon‐black‐modified (left image), pristine (middle image), and Zn‐foil‐wrapped cotton yarn (right image). b) Digital photograph of the CYSAB. c) Schematic representation of the working principle of the CYSAB. d) Open‐circuit voltage of the CYSAB in response to a 100 µL NaCl solution (80 × 10^−3^ m). e) Polarization curves of the CYSAB as the concentration of the NaCl solution is increased from 20 × 10^−3^ to 320 × 10^−3^ m. f) Power density curves of the CYSAB as the concentration of the NaCl solution is increased from 20 × 10^−3^ to 320 × 10^−3^ m. The data obtained from three independent experiments (*n* = 3) are presented as the mean ± standard deviations.

Carbon‐based materials have been widely used as efficient metal‐free electrocatalysts for O_2_ reduction in metal‐air batteries.^[^ ^41^ ^]^ Due to its low cost, high conductivity, biocompatibility, and great stability, carbon black was chosen as the cathode material to construct the CYSAB via a layer‐by‐layer coating method with bovine serum albumin (BSA) as a binder (see Figure S2b in the Supporting Information).^[^ ^42^, ^43^ ^]^ During the fabrication process, the successively dropped BSA solution and carbon black suspension were absorbed into the gaps between the cotton fibers, forming a tightly attached conductive layer. In order to improve the wettability of the modified yarn, sodium dodecyl benzene sulfonate (SDBS), an anionic surfactant, was utilized to pretreat carbon black powders (see Figure S2a,c in the Supporting Information). The carbon‐black‐coated yarn exhibited good wettability and an electrical conductivity of 59.7 ± 6.3 Ω cm^−1^ (see Figure S3 in the Supporting Information). In comparison to the smooth fiber surface of pristine yarn, a dense layer of micro‐ and nanosized particles was uniformly distributed on the modified fibers, suggesting the successful fabrication of a yarn‐based cathode segment (**Figure** **2**a). After carbon black immobilization, the color of the cotton yarn changed from light yellow to black (Figure 2b). The interlaced fibers and surface‐packed particles endowed the cathode segment with a hierarchically porous structure, which may facilitate the transfer and reduction of oxygen molecules (see Figure S4 in the Supporting Information). A long cathode could provide a large surface area for oxygen adsorption and abundant active sites toward oxygen reduction. We fabricated devices with the cathode length ranging from 4 to 10 cm. It was observed that the CYSAB with the 4 cm cathode exhibited the highest voltage (see Figure S5 in the Supporting Information). Therefore, we selected 4 cm as the optimal cathode length to fabricate devices.

Considering the CYSAB anode, Zn foil was wrapped around the yarn to achieve tight contact with the fibers (see Figure 2a and Figure S6 in the Supporting Information). It should be noted that Zn foil could be spontaneously oxidized into ZnO in ambient environment, forming a surface layer with the thickness of tens of nanometers. The biocompatibility of ZnO to human skin has been confirmed in a lot of previous studies.^[^ ^44^ ^]^ ZnO particles have also been widely used in commercially available sunscreen products.^[^ ^44^ ^]^ Thus, the Zn foil‐wrapped yarn could safely contact human skin. A segment of the pristine yarn remained between the anode and cathode to function as a salt bridge. Figure 2b shows the design of a typical CYSAB with cathode, salt bridge, and anode lengths of 4.0, 0.5, and 1.0 cm, respectively. Once sufficient sweat has been wicked into the yarn, it quickly flows along the yarn to impregnate the salt bridge, further connecting the cathode and anode. Through this action, ions contained in sweat start to migrate between the two electrodes, and the battery is activated. As shown in Figure 2c, the reduction reaction (O_2_ + 2 H_2_O + 4 e^−^→ 4 OH^−^) occurs at the cathode, while the oxidation reaction (Zn – 2e^−^→ Zn^2+^) occurs at the anode. Electrons generated by the anode flow to the cathode through an external circuit, inducing a constant DC output current.

Because Na^+^ and Cl^−^ are the major ions in sweat, an 80 × 10^−3^ m NaCl solution was employed to mimic human sweat for the activation of the fabricated CYSAB.^[^ ^22^ ^]^ Upon dropping a 100 µL NaCl solution at the salt bridge position, a sharp enhancement in the open‐circuit voltage (*V*_oc_) was observed, reaching a stable plateau at ≈1.0 V in 5 s (Figure 2d). Polarization and power density curves were obtained to investigate the effects of the NaCl concentration on the CYSAB (Figure 2e,f). As the NaCl concentration increased from 20 × 10^−3^ to 320 × 10^−3^ m, the slopes of the corresponding polarization curves in the Ohmic region significantly decreased, suggesting a decrease in the internal electrical resistance of the battery. Because the ionic conductivity of the electrolyte is proportional to the NaCl concentration, the infiltration of an identical volume of a concentrated NaCl solution may lead to the CYSAB exhibiting a low internal resistance. The maximum power density of the device is also linearly correlated with the NaCl concentration of the electrolyte in the range of 20 × 10^−3^ to 320 × 10^−3^ m (see Figure S7 in the Supporting Information). The CYSAB filled with 100 µL of 20 × 10^−3^ m NaCl solution delivered a maximum power density of 6.4 µW cm^−2^. However, as the concentration increased to 320 × 10^−3^ m, the peak power density reached 33.1 µW cm^−2^, which is almost five times higher than that obtained with the 20 × 10^−3^ m NaCl solution. When an external load of 50 kΩ was connected in series with the CYSAB filled with 100 µL of 80 × 10^−3^ m NaCl solution, the output voltage and output current could be maintained at ≈0.4 V and ≈8 µA over 1 h, respectively (see Figure S8 in the Supporting Information). Based on the data, it can be observed that a higher NaCl concentration induces a better performance. Because our battery is designed as a sweat‐activated device, the concentration of ions contained in human sweat should also be considered in the performance evaluation of CYSABs. Normally, concentrations of Na^+^, Cl^−^, K^+^, and Ca^2+^ in human sweat are in the ranges of 10 × 10^−3^ to 100 × 10^−3^, 10 × 10^−3^ to 100 × 10^−3^, 1 × 10^−3^ to 18.5 × 10^−3^, and 0.41 × 10^−3^ to 12.4 × 10^−3^ m, respectively.^[^ ^40^ ^]^ Considering the existence of other ions, the total ion concentration of sweat should not exceed 320 × 10^−3^ m. Therefore, the NaCl concentrations tested in the present study could represent the ionic strength in human sweat for estimating the battery power density required for practical applications.

**Figure 3:**
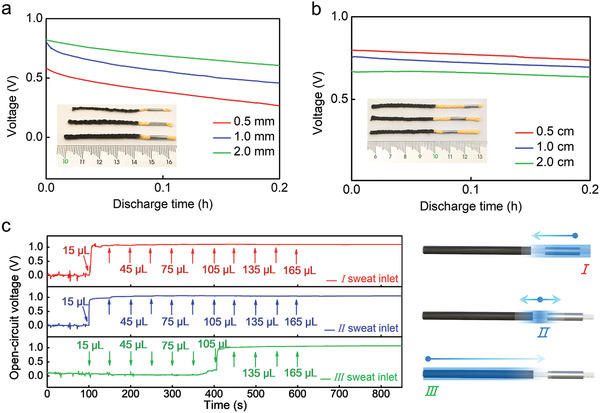
a) Galvanostatic discharge curves of CYSABs with the yarn diameters of 0.5, 1.0, and 2.0 mm, respectively. b) Galvanostatic discharge curves of CYSABs with salt bridge lengths of 0.5, 1.0, and 2.0 cm, respectively. Insets in (a) and (b): digital photographs of the corresponding devices. c) Effects of the sweat inlet location and volume on the starting time of the CYSABs.

To optimize the CYSAB performance, we investigated the influence of the yarn diameter and length of the salt bridge segment on the galvanostatic discharge behavior. As shown in **Figure** **3**a, the battery fabricated with a 2.0 mm thick yarn had a higher voltage during the discharge process in comparison to the devices prepared with fine yarns (diameter = 1.0 and 0.5 mm). A thicker yarn may possess more capillary channels to facilitate sweat transport and ionic migration, resulting in a device with a higher output voltage. The length of the salt bridge can also significantly affect the performance of the CYSAB. As the salt bridge length increased from 0.5 to 2.0 cm, the output voltage remarkably decreased during the discharging process (Figure 3b). Reducing the salt bridge length to 0.3 cm could not further enhance the performance of CYSAB (see Figure S9 in the Supporting Information). Considering the availability of yarn and the difficulty of fabrication, we chose a 2.0 mm thick cotton yarn to prepare CYSABs with a salt bridge length of 0.5 cm in the following assays.

Sweat infiltration is essential for the activation of yarn‐based batteries. Cotton yarn was chosen as the substrate material for 1D SAB fabrication because of its high hydrophilicity and excellent water storage capability. By weighing the mass enhancement of the devices, it was found that the as‐prepared CYSAB could store 160.3 ± 13.2 µL of the 80 × 10^−3^ m NaCl solution in its saturated state (see Figure S10 in the Supporting Information). The CYSAB could only be triggered when the sweat electrolyte passed through the salt bridge segment to effectively connect the anode and cathode (Figures 1c and 2c). Because the perspiration rate is closely related to the individual, physical conditions, body location, and surrounding environment, the sweat volume and inlet location on the device should be studied in the battery activation process. As illustrated in Figure 3c, an 80 × 10^−3^ m NaCl solution was dropped onto the CYSAB from locations I (anode side), II (salt bridge segment), and III (edge of the cathode) several times with a volume of 15 µL. From the *V*_oc_–*t* curves, it can be observed that 15 µL of the NaCl solution could initiate the battery when added from locations I and II. The addition of more electrolyte solution did not induce an obvious change in *V*_oc_. Although 15 µL of electrolyte is sufficient to switch on the battery in both cases, the response time for the device with the inlet at location I is 6.8 s, which is slightly longer than that with the inlet at location II (3.2 s). The electrolyte solution absorbed at the salt bridge component (0.5 cm in length) rapidly flows to both sides along the yarn to link the anode and cathode. However, the anode‐side‐absorbed electrolyte needs to pass through the Zn‐wrapped section (1.0 cm in length) and salt bridge segment (0.5 cm in length), resulting in a late response. In contrast to the above cases, the volume for CYSAB activation increased to 105 µL when the NaCl solution infiltrated from location III at the cathode edge. In this case, the electrolyte needs to flow through the 4 cm carbon‐black‐modified and 0.5 cm salt bridge segments. The dead volumes of these two segments and the blockage effects caused by carbon black may cause an enhancement in the activating volume. It has been reported that the rate of perspiration may reach 6.0, 4.0, and 1.5 µL min^−1^ cm^−2^ at the human forehead, chest, and arm, respectively.^[^ ^45^, ^46^ ^]^ Therefore, our CYSAB could be activated in a few minutes if skin secreted sweat is absorbed by this device from the appropriate inlet locations.

**Figure 4:**
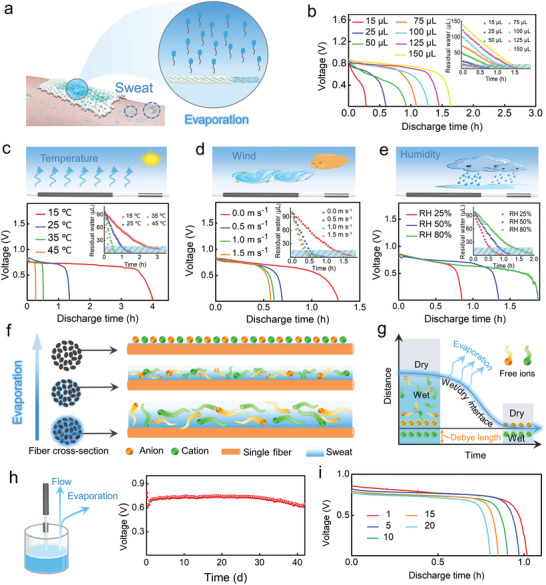
a) Schematic illustration of water evaporation from a CYSAB. b) Galvanostatic discharge curves of CYSABs infiltrated with different volumes of the NaCl solution under ambient conditions. Effects of c) temperature, d) wind, and e) humidity on the galvanostatic discharge curves of CYSABs infiltrated with 100 µL of 100 × 10^−3^ m NaCl solution. The insets in (b)–(e) show the water evaporation curves of the devices infiltrated with 100 µL of 100 × 10^−3^ m NaCl solution. f) Schematic illustration of ion navigation in the water layer formed on the fiber surface. g) Schematic illustration of the mechanism for the reduction of ionic mobility during water evaporation. h) Long‐term galvanostatic discharge curve of a CYSAB with a continuous electrolyte supply. i) Reusability of a CYSAB.

Although a certain amount of electrolyte solution could be reserved in the cotton yarn to sustain the battery operation, water evaporation from the yarn is inevitable, resulting in device failure (**Figure** **4**a). Under ambient conditions (*T* = 25 °C, relative humidity = 65%), a CYSAB infiltrated with 100 µL of the NaCl solution from the anode side could maintain a constant *V*_oc_ of ≈1.0 V for ≈1 h, after which *V*_oc_ gradually decreased to zero (see Figure S11 in the Supporting Information). The water evaporation from the device should be the reason for the *V*_oc_ reduction observed in this experiment. The galvanostatic discharge curves of CYSABs containing different volumes of the NaCl solution were recorded to further investigate their performances in daily environments (Figure 4b). As indicated by the findings shown in Figure 3c, 15 µL of the NaCl solution could trigger the battery to produce electricity. However, at a discharge current of 15.9 µA cm^−2^, the output voltage decreased very rapidly, finally dropping to zero after only 17 min. When the volume of the electrolyte solution increased to greater than 25 µL, constant output voltages could be obtained. As the electrolyte volume increased from 25 to 150 µL, the discharge voltage plateau gradually increased to ≈0.8 V. The initial discharge voltage also exhibited the same trend (see Figure S12 in the Supporting Information). The presence of more electrolyte solution on the yarn may be conducive to achieving charge equilibrium between the two electrodes, further leading to the enhancement of the discharge voltage plateau. Moreover, the discharge time increased from 17 min to 1.6 h as the electrolyte volume increased. Because the output voltage continuously decreased for the device containing 15 µL of the NaCl solution without forming a discharge voltage plateau, 15 µL was set as the failure volume of the CYSAB to analyze the evaporation curves (see the inset in Figure 4b). Interestingly, the time corresponding to 15 µL matches well with the duration of the discharge voltage plateau, suggesting the critical role of water evaporation in the lifetime of the CYSAB. The mass change of the CYSABs that absorbed different volumes of the NaCl solution was monitored to estimate the water evaporation from the devices. It was observed that the devices containing more electrolyte required longer times to reach the failure volume, thereby providing a longer discharge duration.

In practical applications, water evaporation can be affected by environmental parameters, such as temperature, wind, and humidity. Therefore, the water evaporation and performance of the CYSABs infiltrated with 100 µL of the NaCl solution were measured at different temperatures, wind speeds, and humidities. Similar to the findings shown in Figure 4b, 15 µL could be used as the water‐remaining threshold to estimate the duration of the discharge voltage plateau in the assays. An increase in temperature in the range of 15 to 45 °C accelerated the evaporation from these devices, leading to a reduction in the discharge time (Figure 4c). The increase in wind speed also induced the same trend. Faster evaporation and shorter discharge times were achieved for the batteries exposed to higher wind speeds (Figure 4d). As the relative humidity increased from 25% to 80%, water evaporation was inhibited, further causing the elongation of the battery discharge process (Figure 4e). Based on the above results, it can be concluded that the evaporation of the yarn‐absorbed electrolyte solution could lead to battery failure.

Electricity generation from the CYSAB greatly relies on the wicking of the electrolyte solution into capillary channels between the fibers and the migration of ions in the aqueous environment (Figure 4f,g). According to the Stern model, when the cotton fiber surface is in contact with the electrolyte solution, an electrical double layer (EDL) forms at the solid–liquid interface, which consists of the Stern and diffuse layers.^[^ ^47^, ^48^ ^]^ The movement of ions in the EDL is restricted by the electric forces, and the EDL thickness can be defined using the Debye length (*λ*_d_).^[^ ^49^ ^]^ When the NaCl solution is added, the gaps between the fibers are filled with the electrolyte. The thickness of the liquid layer on the fiber surface is much larger than *λ*_d_. Therefore, the ions can freely migrate in the region far from the EDL, enabling the efficient conversion of chemical energy into electricity in the battery. As water evaporates from the device, the amount of yarn‐absorbed solution gradually decreases. The thickness of the liquid layer on the fiber surface continuously approaches *λ*_d_. Once it decreases to a value close to *λ*_d_, most of the ions in the electrolyte are confined in the EDL region, which significantly affects the charge transfer between the anode and cathode. Battery performance is significantly impaired under these conditions. Finally, the CYSAB stops working because of water exhaustion. Therefore, it is crucial to maintain the amount of electrolyte solution in the device to achieve a long‐term supply of electric power.

Because yarn can uptake water via capillary effects without an external pump, CYSABs with a continuous electrolyte supply may be prepared by immersing one end of the yarn in a NaCl solution. Although water evaporation from the device still occurs, the evaporated water is displaced by the wicking of fresh electrolyte. As shown in Figure 4h, the output voltage from a CYSAB with the continuous electrolyte supply was maintained at a very stable level for more than 40 d (*T* ≈ 25 °C, RH ≈ 70%). It should be noted that the evaporation‐induced “off‐state” of the battery could also be very useful in practical applications. Because perspiration usually occurs during exercise, CYSABs can be used to power monitoring devices only during exercise. The system could be spontaneously powered off after usage and recovered to its “on‐state” during the next round of exercise. Figure 4i demonstrates the recovery of a CYSAB in the evaporation‐induced “off‐state” upon the addition of a NaCl solution. Even after 20 evaporation cycles, the battery could still be initiated by the electrolyte solution.

**Figure 5:**
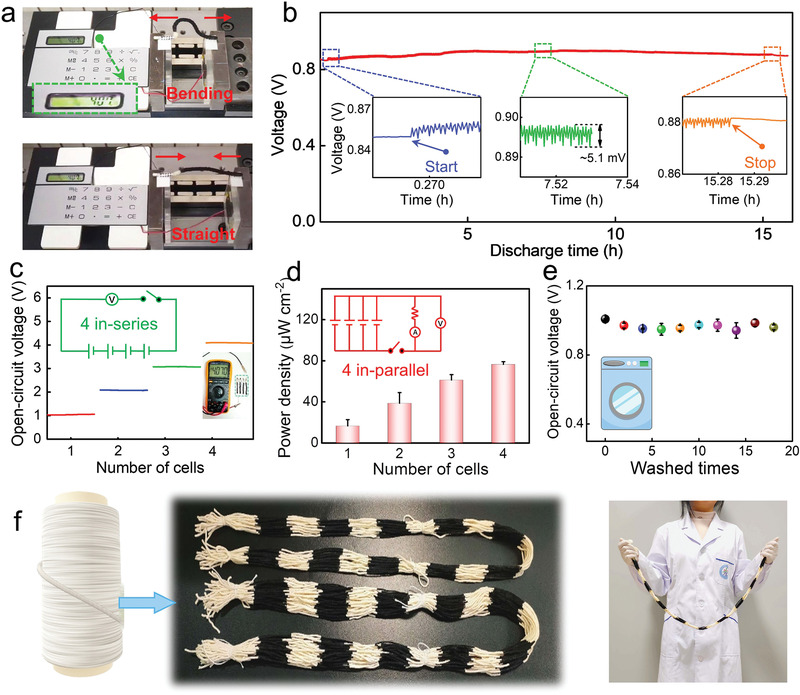
a) Calculator powered by a CYSAB during repeated bending. b) The galvanostatic discharge curve of a CYSAB under repeated bending at a frequency of 0.5 Hz over 20 000 cycles. c) Open‐circuit voltages of series‐connected battery packs. Insets: photos of a multimeter during the real‐time measuring of a series‐connected battery pack with 4 CYSABs. d) Power densities of parallel‐connected battery packs. e) Effect of repeated washing on the open‐circuit voltage of a CYSAB. The data obtained from three independent experiments (*n* = 3) are presented as the mean ± standard deviations. f) Scale‐up production of long yarns with several cathode segments.

A single device was fixed to a stepping motor for a flexibility test. Upon the addition of 100 µL of the NaCl solution, the device could produce electricity to power a calculator in both the straight and bending states (**Figure** **5**a). As clearly demonstrated in Video S1 in the Supporting Information, the calculator could work normally even when the battery experienced repeated bending at a frequency of 1 Hz. The galvanostatic discharge curve illustrated that the repeated bending of the activated CYSAB only induced a slight reduction on its output voltage (see Figure S13a and Video S2 in the Supporting Information). Moreover, the device with continuous NaCl solution supply could tolerate up to 20 000 times of bending without significant drop on its output voltage (Figure 5b). Once the bending stops, the output voltage immediately returns to its original level. These results verify that the CYSAB activated with the electrolyte could tolerate repeated bending to deliver adequate electricity to power miniaturized electronic devices.

Several CYSABs can be connected in series or parallel to form battery packs. As the number of in‐series connected CYSABs increased from 1 to 4, the *V*_oc_ of the battery pack increased from 1.03 to 4.07 V (Figure 5c). Considering parallel‐connected battery packs, the power density linearly increased with the increase in the number of batteries (Figure 5d). Under a load of 10 kΩ, the output voltage and output current of a single CYSAB were 285.6 mV and 26.9 µA, respectively. For a pack containing four in‐parallel batteries, these values increased to 673.5 mV and 73.1 µA, respectively (see Figure S14a in the Supporting Information). Two series‐connected CYSABs could be employed to decorate a patterned cloth for powering a red light‐emitting diode (LED) with a minimum driving voltage of 1.5 V (see Figure S14b in the Supporting Information). These findings indicate the good voltage, current, and power scalability characteristics of the CYSABs when connected in series and parallel configurations.

A CYSAB was washed with water containing 1 wt% detergent under stirring for 10 min, followed by air drying under ambient conditions. The *V*_oc_ of the battery activated with a 100 µL NaCl solution was examined after each washing–drying process. There was no significant change in the *V*_oc_ of the device after 16 washes. The surface morphology of the carbon‐black‐coated part was also characterized before and after washing, showing that most of the coating layer could be preserved after 16 times of washing (see Figure S15 in the Supporting Information). Both *V*_oc_ data and scanning electron microscope images indicate the excellent washability of this device (see Figure 5e and Figure S16 in the Supporting Information). Therefore, the developed CYSAB is a very promising 1D energy source with great flexibility and washability for textile‐based wearable systems.

The long‐length yarn containing CYSABs is of great importance for the fabrication of electronic textiles using conventional weaving techniques. In the present study, the segmented structure of the CYSAB was adopted to prepare multiple batteries on a long yarn. As shown in Figure 5f and Figure S17 in the Supporting Information, 10 carbon‐black‐based cathodes could be successfully fabricated on a 1.0 m long yarn at predesigned locations. Moreover, this approach could be scaled‐up to produce bundles of long‐length yarns. In such a multiple‐in‐one structure, a segment of the pristine yarn serves as the linking component between two neighboring CYSABs. Upon infiltration of the electrolyte solution, the liquid and ions may be transferred along the linking segment between adjacent batteries, resulting in a performance reduction. To avoid the adverse effects, the linking segments were modified using a hydrophobic material to form water barriers, which could completely block the transfer of the electrolyte and ions between adjacent batteries in the multiple‐in‐one device structure (see Figure S18a,b in the Supporting Information). Two energy yarns consisting of two series‐connected CYSABs with/without a hydrophobic barrier were fabricated for performance comparison. The output voltage of the device with the hydrophobic barrier was 1.25 V, which is two times higher than that without the barrier (see Figure S18c in the Supporting Information). The as‐prepared energy‐producing yarn could be worn on the wrist of a volunteer to power an LED display timer (see Figures S18d and S19 in the Supporting Information).

**Figure 6:**
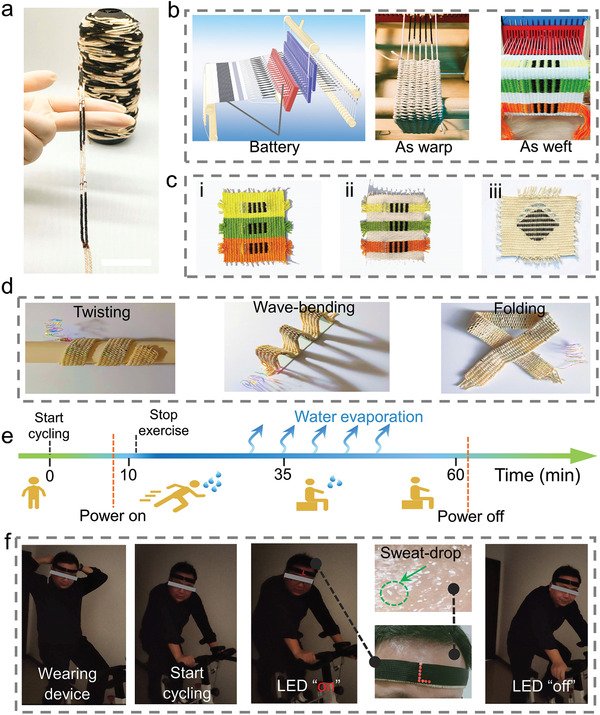
a) Photograph of a spindle of yarn containing multiple CYSABs; b) sweat‐activated energy fabrics (SAEFs) woven with the energy‐producing yarn as warps or wefts. c) Photographs of the SAEFs with different patterns. d) Photographs of a set of LEDs powered by the SAEF under twisting, wave‐bending, and folding states. e,f) On‐body demonstration of self‐powered headlights mounted on an SAEF‐based headband during exercise.

Thus far, the SABs have mainly been fabricated using paper as the sweat absorption layer and electrode spacer, which may limit the large‐scale production of SABs and their applications in textile electronics. The 1D design of CYSABs enables their easy integration into electronic fabrics via conventional textile techniques such as weaving, knitting, sewing, and stitching. **Figure** **6**a shows a spindle of energy‐producing yarns containing multiple CYSABs, which can be used as warps or wefts to weave fabrics. In this study, a simple frame loom was utilized to weave plain fabrics with CYSABs (Figure 6b and Figure S20 in the Supporting Information). Because of the design flexibility of the woven structure, the distribution of CYSABs on the fabrics could be tailored to generate different patterns, thereby meeting aesthetic requirements (Figure 6c and Figure S20 in the Supporting Information). The energy yarns containing five series‐connected CYSABs were used as warps to weave a strip of sweat‐activated energy fabric (SAEF), in which six strings of series‐connected batteries were connected in parallel to form a battery pack. Owing to the woven structure, SAEFs possess excellent mechanical flexibility, endowing the fabricated devices with high conformability to curved surfaces. After absorption of the NaCl solution, the SAEF strip could power multicolor LEDs in the twisted, wave‐bent, and folded states (Figure 6d). To demonstrate the feasibility of these devices, a self‐powered headlight mounted on an SAEF‐based headband was prepared by connecting the LEDs in series with the pack of CYSABs. Using the homemade headband on the forehead of a volunteer, the volunteer began cycling on a spin bicycle. After ≈8 min of exercise, the volunteer started sweating. Further exercise induced the secretion of sweat from the skin to form sweat droplets on the forehead. The sweat was absorbed by the headband to activate the CYSABs, consequently switching on the LEDs (see Figure 6e,f and Video S3, Supporting Information). Interestingly, the LEDs on the headband remained on 1 h after exercise, indicating that the fabric could serve as a sweat reservoir to support continuous electricity output from the CYSABs. The CYSAB and SAEF may be integrated with gears to trigger safety warning lights for night jogging or night cycling.

**Figure 7:**
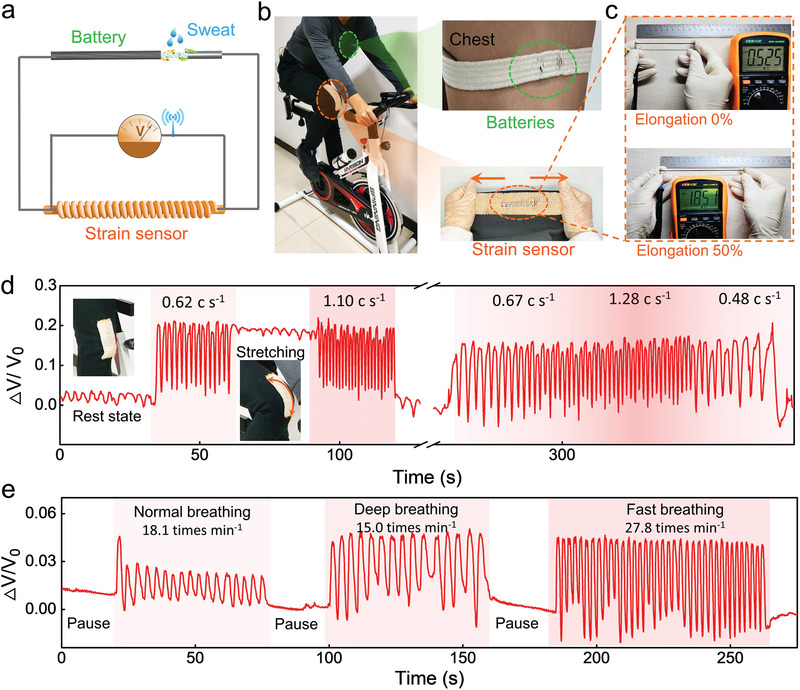
a) Equivalent electric circuit of a self‐powered system containing cotton‐yarn‐based sweat‐activated batteries and elastic‐yarn‐based strain sensors. b) Integration of a sweat‐activated energy fabric with a fabric‐based strain sensor for on‐body tests. c) Resistance variation of an elastic‐yarn‐based strain sensor under 0% and 50% strains. Self‐powered real‐time monitoring of d) cycling velocity and e) breathing rate for a volunteer while cycling with the strain sensors located at the knee and abdomen, respectively.

To further demonstrate the potential of SAEFs, a yarn‐shaped strain sensor was woven into a fabric and integrated with an SAEF to construct a self‐powered sensing system (**Figure** **7**a,b). The strain sensor was fabricated by dipping a core‐sheath‐structured elastic yarn into a conductive ink consisting of poly(3,4‐ethylenedioxythiophene):poly(styrenesulfonate) (PEDOT:PSS) and carbon black powder (75 wt%:25 wt%). After modification, a dense conductive layer was successfully coated on the fibers in the sheath without affecting the elastic core (see Figure S21 in the Supporting Information). Under a strain of 50%, the resistance of the elastic yarn changed from 0.53 to 1.85 MΩ (Figure 7c), which may be caused by the structural change of the sheath from dense to loose during the stretching process (see Figure S21a in the Supporting Information). The resistance change of the conductive yarn can be utilized to monitor human body motions and vital signs. The stretchable fabric containing the strain sensor was fixed at the knee and abdomen of a volunteer for the real‐time monitoring of knee bending behaviors and breathing patterns during exercise, respectively (see Figures S23 and S24 in the Supporting Information). A strip of the SAEF worn on the volunteer's chest was connected to the sensing fabric to provide an energy supply. Once the SAEF absorbed enough sweat, the system began to function. The alteration of the sensor resistance leads to a change in the output voltage according to the following equation

(1) $$V_{\mathrm{Output}}=V_{\mathrm{OC}}\frac{R_{\mathrm{Sensor}}}{R_{\mathrm{Internal}}+R_{\mathrm{Sensor}}}=V_{\mathrm{OC}}\frac{1}{\frac{R_{\mathrm{Internal}}}{R_{\mathrm{Sensor}}}+1}$$

where *V*_Output_ is the output voltage, *R*_Internal_ is the internal resistance of the SAEF, and *R*_Sensor_ is the resistance of the strain sensor. Therefore, the change in the output voltage was recorded in real time to evaluate human motion and breathing.

During cycling, the volunteer's knee was repeatedly bent and straightened, simultaneously driving the stretching and release of the fabric strain sensor. As illustrated in Figure 7d and Video S4 in the Supporting Information, the real‐time measured curve could be divided into several stages. In the first stage, the volunteer was resting on the bicycle, keeping the knee in a straight state. Because there is no strain on the sensor, a stable output voltage can be observed. In the second stage, the volunteer starts cycling, and the motion of the volunteer's knee induced the periodic oscillation of the output voltage. The frequency of the oscillation could be used to estimate the cycling speed at 0.62 cycle s^−1^. In the third stage, the knee stopped at the bending state, resulting in the stretching of the sensor. In comparison to the original resting state, the output voltage remained stable at a higher level. The volunteer then started cycling again at a higher speed. Signals with higher frequencies could be detected, demonstrating a speed of 1.10 cycle s^−1^. The change of the cycling speed from 0.67 to 1.28 to 0.48 cycle s^−1^ could also be differentiated from the real‐time curve. In addition to the bending in human knees, periodic oscillation may also occur during the cycling process. In order to investigate the impact of periodic oscillation, the vibration of the ruler was tested with the sensing strip. The ruler‐vibration‐caused voltage changes are less than 1/10 of those at the stretching state (see Figure S24 and Video S5 in the Supporting Information). The signals at the stretching state are at the same level as those induced by knee bending. Therefore, the knee‐induced periodic oscillation does not significantly affect the accuracy of the sensor.

The system could also be used to sense breathing‐induced deformations in the entire abdomen (Figure 7e). When the volunteer held their breath, no change in the output voltage was observed. Normal breathing, deep breathing, and fast breathing led to different output voltage profiles. According to the strength and frequency of the signals, the breathing depth and respiration rate could be determined from the curve. To further confirm the self‐powered capability, a sweat‐activated energy fabric, a strain sensor, and a red LED were connected in series to form a closed loop circuit with no external power supply. The resistance of the strain sensor increased at the stretching state and decreased at the release state, resulting in the turning off and on of the red LED (see Video S6, Supporting Information). The system could also be utilized to reflect the knee bending during the cycling (see Video S6, Supporting Information). The results clearly indicate that CYSABs could be easily integrated into textiles via weaving to provide a reliable energy supply for textile‐based electronic devices.

In comparison to the 2D SABs reported previously, the as‐prepared battery possesses better weavability, washability, and reusability (see Table S1 in the Supporting Information). Because of its 1D structure, the CYSAB showed excellent flexibility and could stably supply power at the curl/randomly coiled states (see Figure S29 and Video S7 in the Supporting Information). Moreover, owing to the great compatibility with the conventional textile industry, CYSABs can be manufactured in the form of yarn and fabric, which may be more acceptable to the public. The directional liquid transport along the yarn should be another superiority of the CYSABs over the 2D devices based on paper or textile. As shown in Figure S30 in the Supporting Information, three CYSABs were interwoven with two cotton yarns to mimic the structure of a plain fabric. As a dyed NaCl solution flowed through the cotton yarns, the CYSABs could be successively wetted through the intersection points. Then, the LEDs connected with the batteries were turning on in a time‐dependent manner. The intersection points of yarns in the woven structure may function as the locations for liquid supply, mixing, and splitting, thus rendering the CYSAB a powerful battery to prepare complex textile electronic systems.

## 3 Conclusions

In this study, we have demonstrated the fabrication and operation of a weavable, washable, and scalable cotton‐yarn‐based Zn‐air battery activated by human sweat. By utilizing the spontaneous liquid absorption and transport properties of cotton yarn, the cotton yarn was used to prepare a sweat‐activated battery consisting of a carbon‐black‐coated cathode, pristine‐yarn‐based bridge, and Zn‐foil‐wrapped anode. Upon the addition of a NaCl solution, the battery could be immediately activated and provide a fairly stable *V*_oc_ of ≈1.0 V. By adjusting the yarn thickness and length of the salt bridge segment, the battery performance could be significantly improved. Because the operation of the fabricated CYSAB relies on the charge transfer capability along the yarn, the output voltage and power density can be greatly affected by the ion concentration and electrolyte volume. Water evaporation from the device may lead to volume reduction, further influencing battery performance. Therefore, the lifetime of the CYSABs could be adjusted by controlling the environmental parameters or designing an electrolyte replenishing unit to meet the requirements of practical applications. The process of evaporation could also be considered to provide an “off” signal to shut down the device for the next round of recovery using sweat. Due to its 1D structure, the CYSAB could be repeatedly bent and washed in water containing 1 wt% detergent without obvious performance loss. Moreover, long‐length yarns containing multiple CYSABs can be manufactured to match the textile techniques. The on‐body trails demonstrate that sweat‐activated energy fabrics woven with series/parallel‐connected CYSABs could be utilized as reliable energy sources to power a headlight and textile‐based strain sensor. This study provides a new design for a 1D yarn‐based SAB that can be perfectly integrated into textile electronics to function as a biocompatible and reliable power source. CYSABs hold considerable promise for the construction of self‐powered textile‐based wearable systems for the monitoring of human motion and healthcare applications.

## 4 Experimental Section

### Materials

Ketjen carbon black (ECP‐600JD) powder was purchased from Guangdong Canrd New Energy Technology Co., Ltd. (Dongguan, China). Copper wires (diameter: 0.2 mm) and zinc foils (thickness: 1.0 mm) were purchased from Qinghexian Jinou Metals Materials Co., Ltd. (Hebei, China). SDBS was purchased from Adams‐beta Co. Ltd. (Shanghai, China). BSA and PEDOT:PSS (concentration = 1.3 wt%) solution were purchased from Sigma‐Aldrich (Shanghai, China). Superhydrophobic paraffin wax was purchased from Long Xiang Beeswax Products Co. Ltd., China. Cotton yarns (diameter = 0.5, 1.0, and 2.0 mm) and elastic yarns (diameter = 1.0 mm) were commercially purchased from a local market in Chongqing, China.

### Sweat‐Activated Battery based on Cotton Yarn

Ketjen carbon black powder (1.0 g) was dispersed in 400 mL of deionized water containing 2.0 g of SDBS under sonication for 1 h. Zn foils were cut into small rectangular pieces with a length of 1.0 cm and a width of 0.7 cm. Cotton yarn was divided into carbon‐black‐coated cathode, salt bridge, and Zn foil‐wrapped anode segments with lengths of 4.0, 0.5, and 1.0 cm, respectively. The carbon black suspension (150 µL) was carefully dropped onto the cathode region and dried in an oven at 80 °C for 30 min. The same region was modified with a 150 µL BSA (0.5 wt%) aqueous solution. The carbon black‐BSA coating cycle was repeated several times to adjust the loading amount of the carbon black powder. The rectangular‐shaped Zn foil is tightly wrapped in the anode region. The melted wax was dropped onto the cotton yarn and cooled down at room temperature for 10 min to obtain a hydrophobic barrier between two neighbored batteries.

For the evaporation test, the electrolyte‐saturated battery was stored in a chamber at various temperatures (15 to 45 °C), humidities (25% to 80%), and wind velocities (0 to 1.5 m s^−1^). The galvanostatic discharge curve was monitored in real time using an electrochemical workstation (CHI760E, Shanghai Chenhua Co. Ltd., China). The effect of mechanical bending was investigated by fixing the device on a linear motor (WMUA512075‐06‐D, China) with repeated bending–stretching cycles (bending diameter *d* ≈ 1.4 cm) at a frequency of 1 Hz.

### Textile Woven with the Cotton‐Yarn‐Based Sweat‐Activated Battery

Using a simple weaving machine, the cotton‐yarn‐based battery was woven into plain‐structured fabrics with various patterns, in which the 1D‐shaped battery could be utilized as either weft or warp. By designing the fabric structure, the number of incorporated batteries could be adjusted. An elastic‐yarn‐based strain sensor could also be integrated into the fabric to prepare sensing textiles for wearable applications.

### Characterization and Measurements

The cross‐sectional morphologies of the Zn‐foil‐ and CB‐coated cotton yarns were characterized using field‐emission scanning electron microscopy (FESEM; JEOL, Tokyo, Japan). The yarn hydrophilicity was tested using a contact angle measurement apparatus (Model JC2000D, Shanghai Zhongcheng Industrial Corp., China) by dropping 2 µL of water on the surface. The potential, current, and resistance of the devices were measured using a Keithley 2450 system (Keithley Instruments Inc., USA). Cyclic voltammetry and galvanostatic charge–discharge measurements were conducted using an electrochemical workstation (CHI 760E). For the washing test, the yarn‐based battery was placed in a beaker filled with water containing 1 wt% detergent under constant stirring for 10 min at 25 °C. Subsequently, the battery was thoroughly rinsed with plenty of water and allowed to naturally air‐dry.

### On‐Body Demonstration

The on‐body tests were approved by the SWU human research ethics committee. All participants provided written, informed consent prior to participation. A volunteer wore a headband that was woven using a group of yarn‐based batteries (six parallel strings of five series‐connected batteries) on his forehead for the on‐body tests. Nine LED lamps were mounted on the headband and connected in series with the batteries to form a circuit. The volunteer exercised on a spinning bicycle. The perspiration of the volunteer and light‐on processes of the LED lamps were captured in real time with a video camera.

A chest belt containing battery packs was worn on the chest of the volunteer and connected in series with a sensing fabric. To obtain self‐powered motion measurements, the sensing fabric was stitched onto the pants at the knee. For self‐powered respiration measurements, the sensing fabric was fixed onto the shirt of the volunteer at the abdomen. During the exercise, the voltage on the sensing device was real‐time measured using an electrochemical station with a wireless signal output (PalmSens4, PalmSens, Netherlands).

### Statistical Analysis

Data in Figures 2e,f and 5d,e were presented as mean ± SD. The sample size (*n*) for each statistical analysis was 3 unless otherwise indicated. The results were tested for difference using Student's *t*‐test with Origin 8.5 software. A level of *p* < 0.05 was considered to be statistically significant.

## Conflict of Interest

The authors declare no conflict of interest.

## Supporting information

Supporting Information

Supplemental Video 1

Supplemental Video 2

Supplemental Video 3

Supplemental Video 4

Supplemental Video 5

Supplemental Video 6

Supplemental Video 7

## Data Availability

Research data are not shared.
