# Supplementary material for: A Weavable and Scalable Cotton‐Yarn‐Based Battery Activated by Human Sweat for Textile Electronics
Source: Adv Sci (Weinh). 2022 Jan 6;9(7):2103822. doi: 10.1002/advs.202103822 (PMC8895049; doi:10.1002/advs.202103822)
Supplement: Supplementary file 1 — Supporting Information [file ADVS-9-2103822-s008.pdf]

WILEY-VCH

ADVANCED SCIENCE
Open Access

Supporting Information

for *Adv. Sci.,* DOI: 10.1002/advs.202103822

A Weavable and Scalable Cotton-Yarn-Based Battery Activated by Human Sweat for Textile Electronics

Gang Xiao, Jun Ju, Hao Lu, Xuemei Shi, Xin Wang, Wei Wang, Qingyou Xia, Guangdong Zhou, Wei Sun, Chang Ming Li, Yan Qiao, and Zhisong Lu**

Supporting Information

A Weavable and Scalable Cotton-Yarn-Based Battery Activated by Human Sweat for Textile Electronics

Gang Xiao, Jun Ju, Hao Lu, Xuemei Shi, Xin Wang, Wei Wang, Qingyou Xia, Guangdong Zhou, Wei Sun, Chang Ming Li, Yan Qiao, and Zhisong Lu**

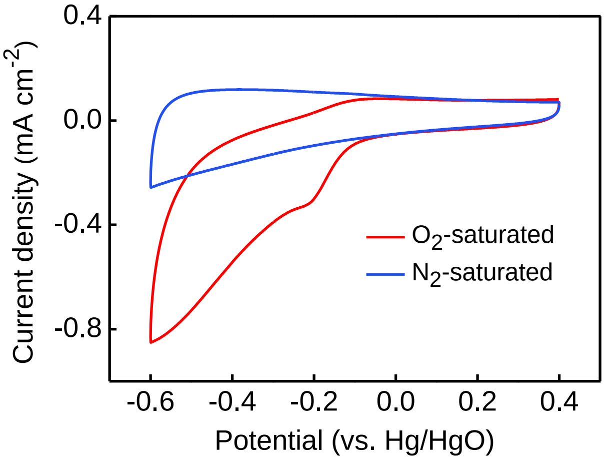

Figure S1. CVs of the carbon-black-modified electrode in N_2-saturated (blue line) and O_2-saturated (red line) NaCl solutions (100 mM) with a scanning rate of 10 mV s^{-1}.

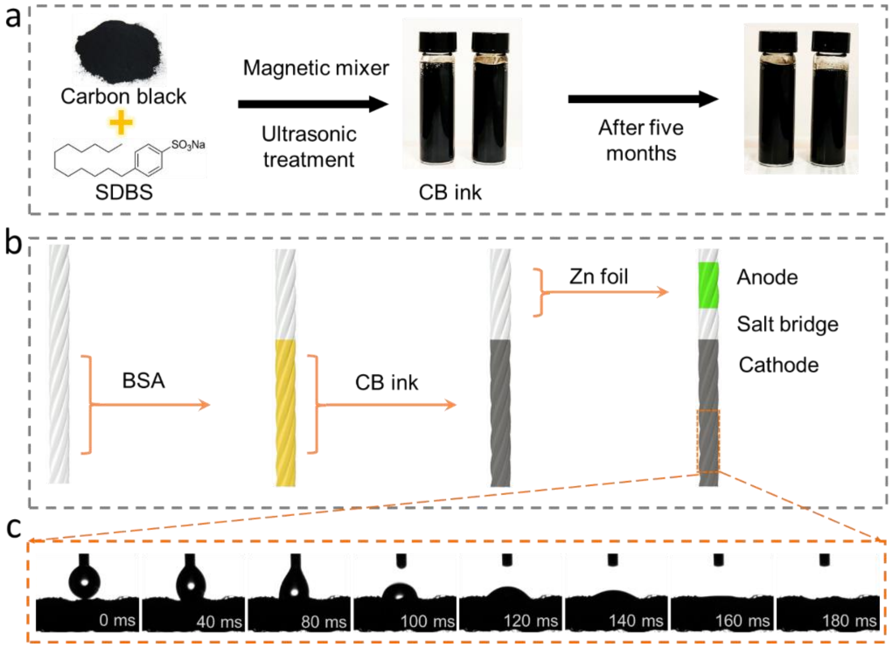

Figure S2. (a) Preparation of carbon black suspension; (b) fabrication of carbon-black- coated cotton yarn with BSA as a binder; (c) contact angle images of the carbon black-coated cotton yarn.

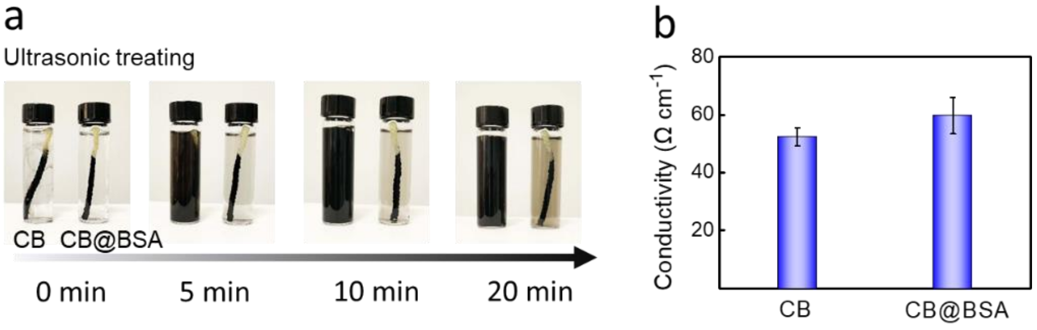

Figure S3. (a) Digital photographs of the carbon-black-modified cotton yarn before and after ultrasonic treatment; (b) conductivity of the carbon-black-modified cotton yarns with/without BSA. The data obtained from three independent experiments (n = 3) are presented as the mean ± SD.

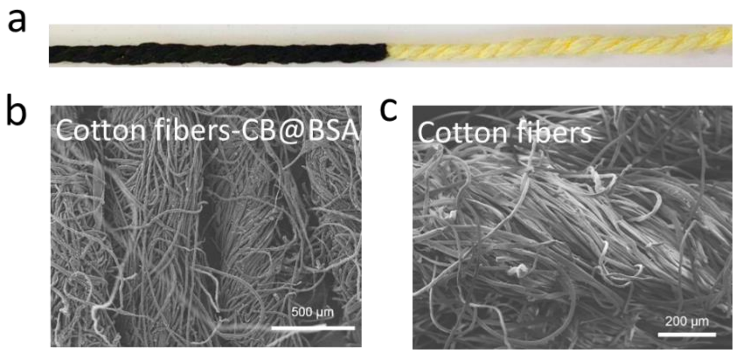

Figure S4. (a) Digital photograph of a cotton yarn partially modified with carbon black; FESEM images of a (b) carbon-black-coated cotton yarn and (c) pristine cotton yarn.

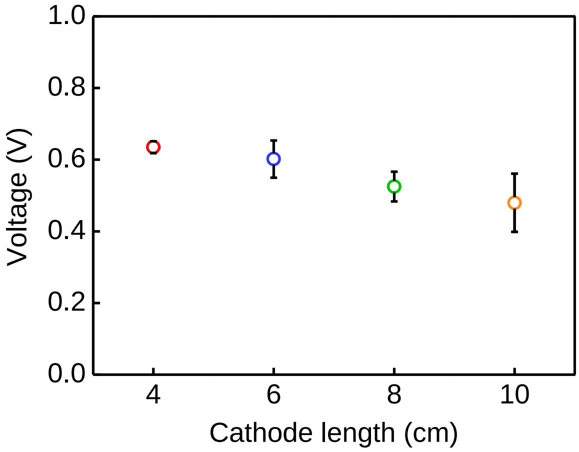

Figure S5. Galvanostatic discharge voltages of CYSABs with the cathode length ranging from 4 to 10 cm. The data obtained from three independent experiments ($n = 3$) are presented as the mean ± standard deviations.

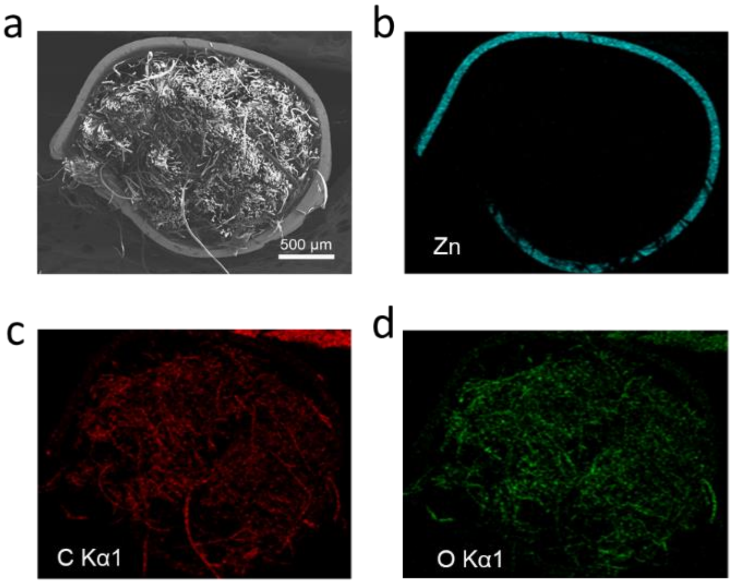

Figure S6. (a) Cross-sectional SEM image of a Zn-foil-wrapped cotton yarn; EDS mappings of Zn-foil-wrapped cotton yarn in terms of the contained (b) Zn, (c) C, and (d) O elements.

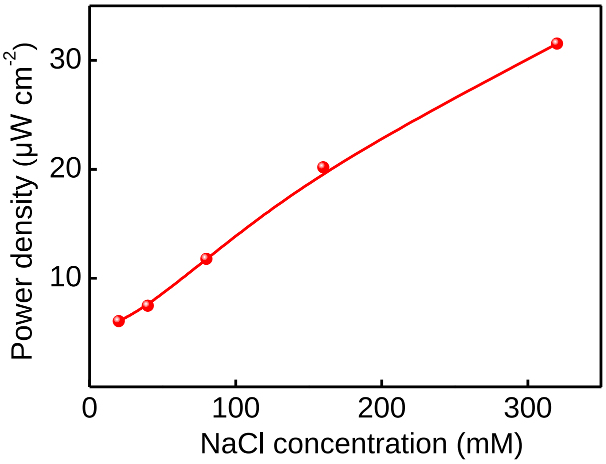

Figure S7. Correlation between the maximum power density of the CYSAB and the NaCl concentration.

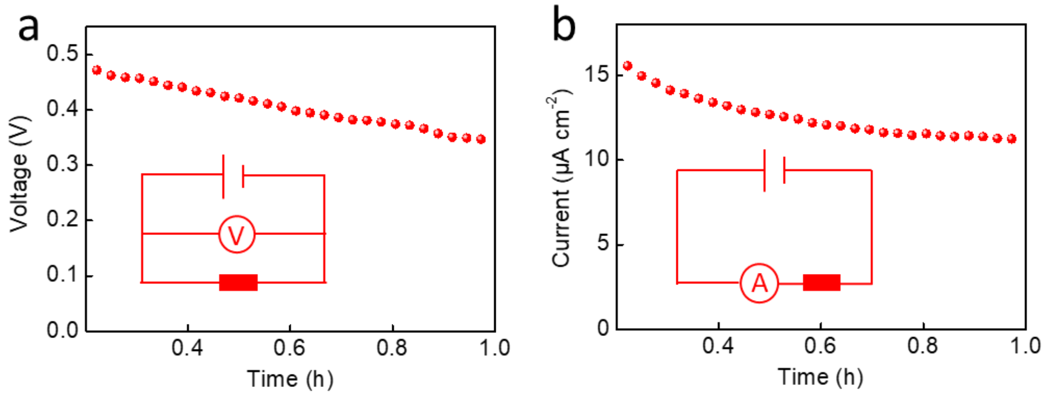

Figure S8. Plots of the (a) voltage and (b) current outputs over time at a given external resistance of 50 kΩ.

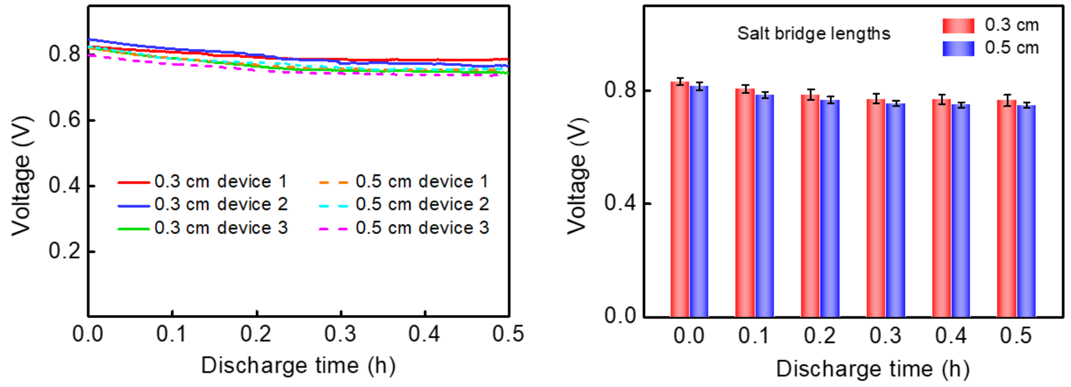

Figure S9. Galvanostatic discharge curves of the devices with the salt bridge lengths of 0.3 cm and 0.5 cm (n=3), respectively.

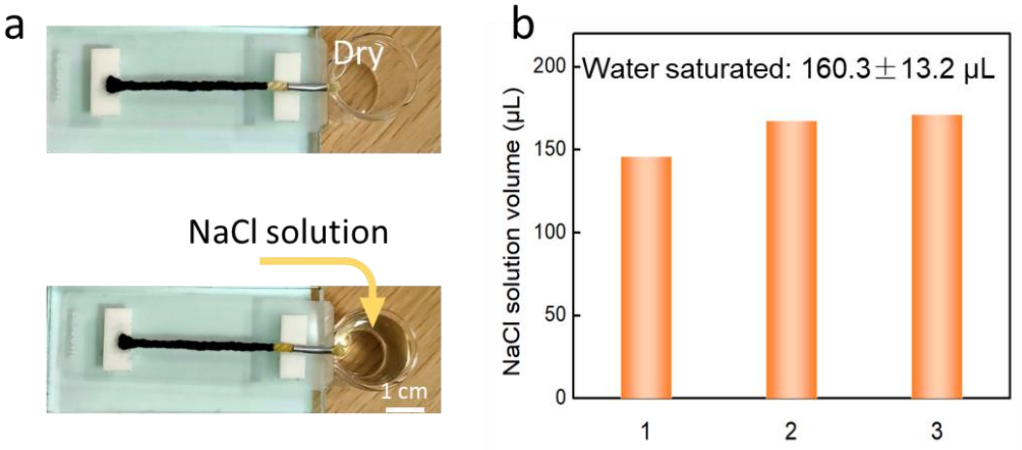

Figure S10. (a) Set-up of device saturation experiments; (b) maximum infiltration volumes of three independent CYSABs (n=3).

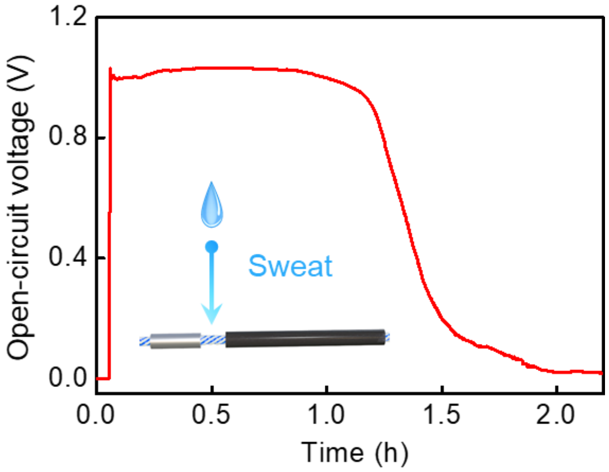

Figure S11. Open-circuit voltage of a CYSAB activated using a 100 μL NaCl solution under ambient conditions.

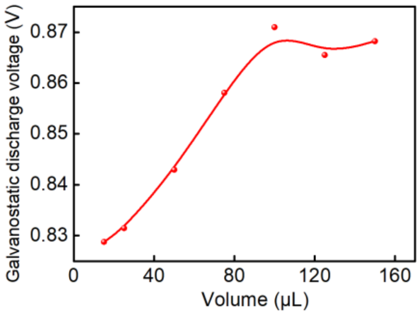

Figure S12. Correlation between the initial voltage in a galvanostatic discharge curve and the volume of a 100 mM NaCl solution.

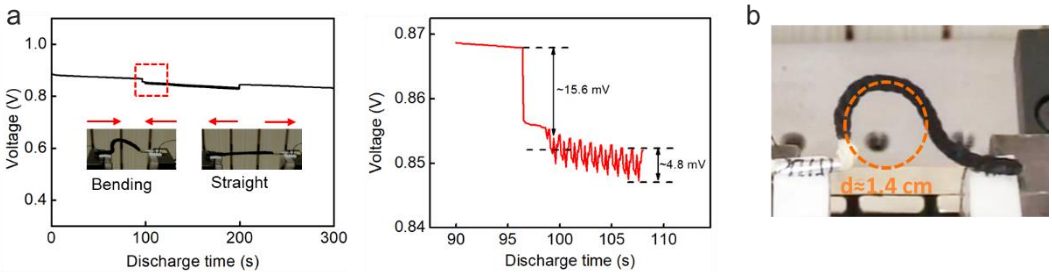

Figure S13. (a) Galvanostatic discharge curve of a CYSAB activated with an NaCl solution under repeated bending. Insets: photographs of a device in bending and straight states, respectively. (b) A device at the bending state. d indicates the bending diameter.

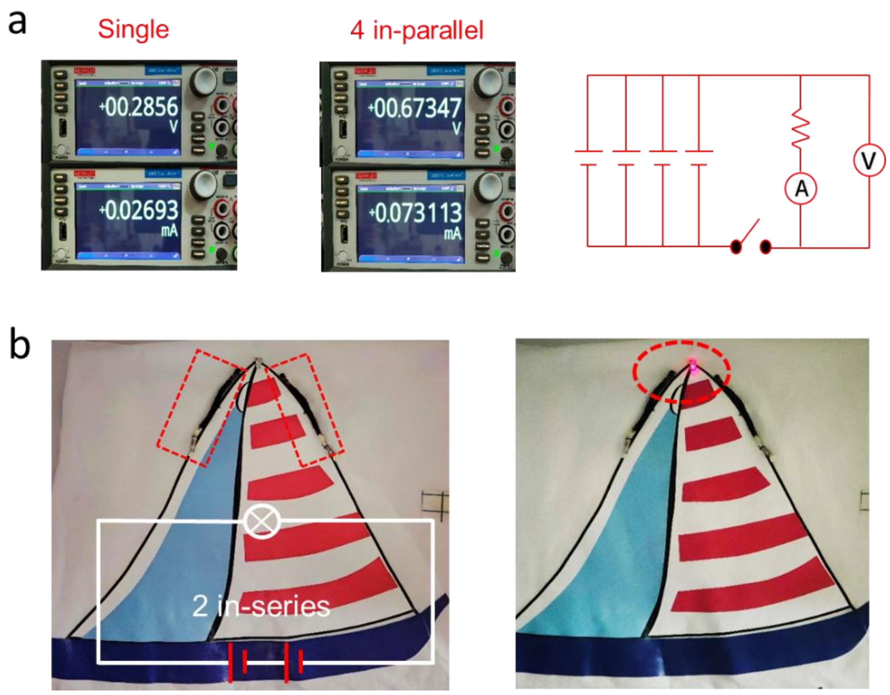

Figure S14. (a) Voltage and current outputs of a battery pack containing four in-parallel CYSABs under a load of 10 kΩ; (b) photograph of a red LED (minimum driving voltage of 1.5 V) powered with two CYSABs connected in series.

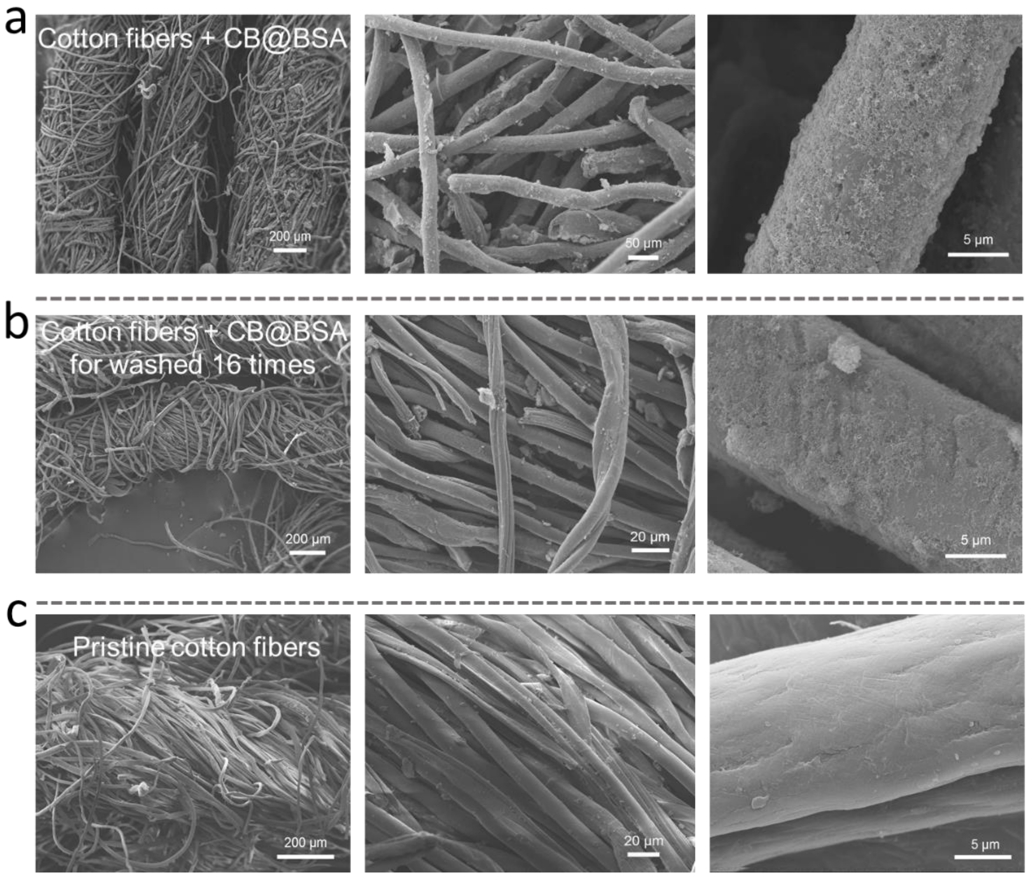

Figure S15. Surface morphologies of carbon-black-coated yarn before (a) and after (b) 16 times of washing. (c) Surface morphology of pristine cotton yarn.

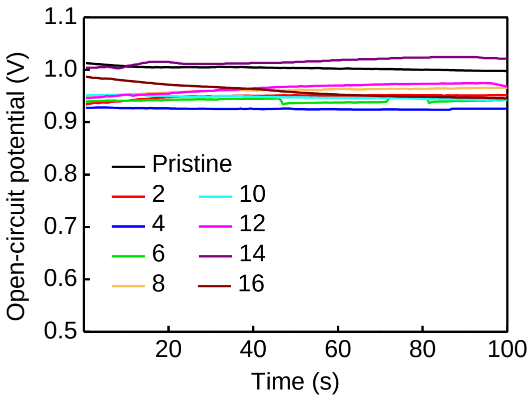

Figure S16. Open-circuit voltage of a CYSAB after repeated washing.

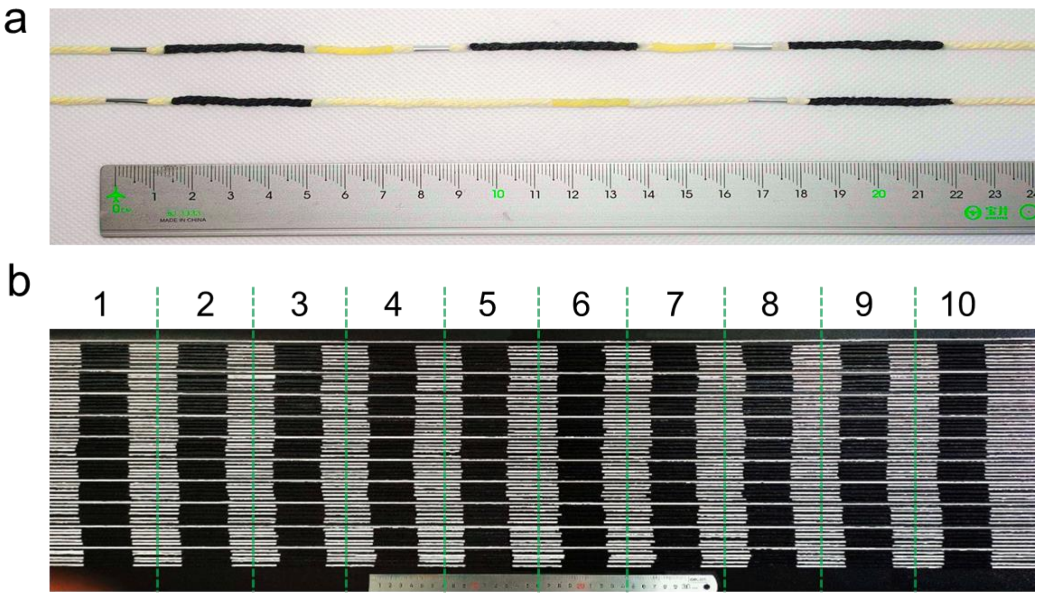

Figure S17. (a) Two cotton yarns with identical lengths containing two (bottom) and three (upper) batteries; (b) photograph showing 50 yarns, each containing 10 continuously fabricated cathode segments.

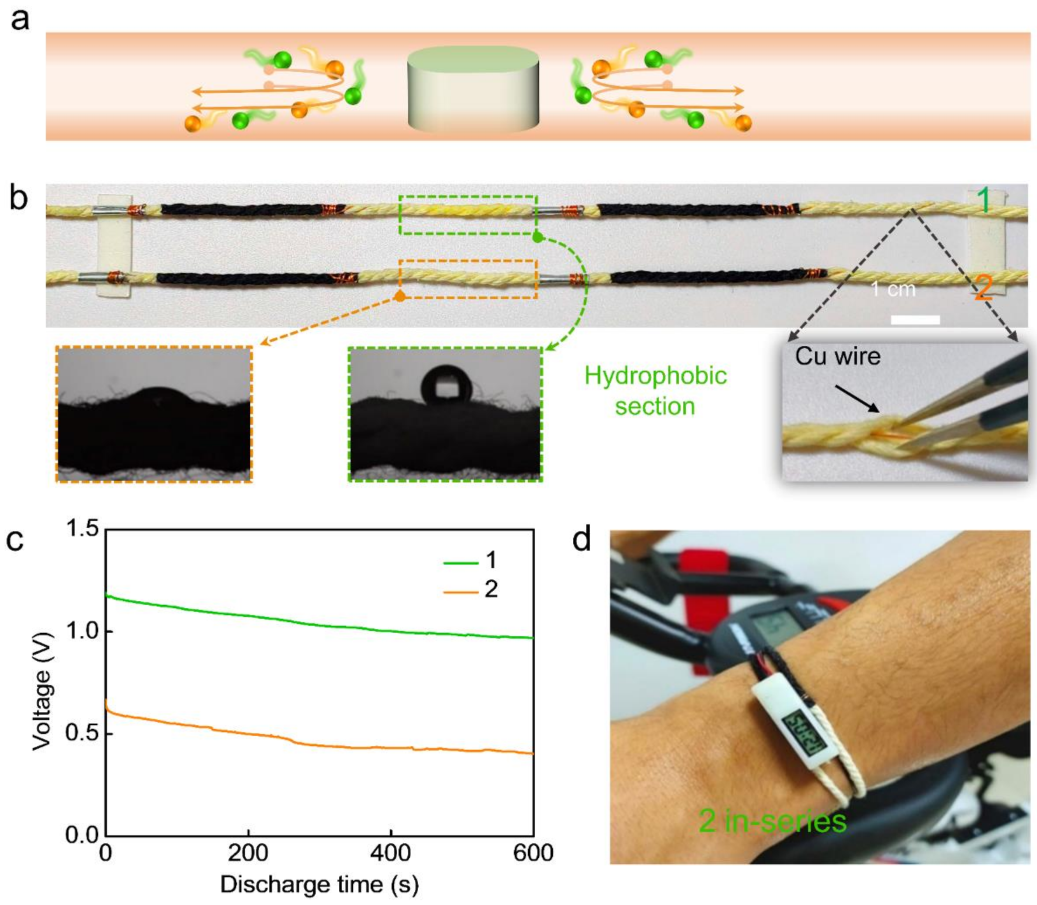

Figure S18. (a) Schematic illustration showing the function of a hydrophobic barrier in a yarn. (b) photographs of two battery packs (two series-connected CYSABs) with (labelled as “1”)/without (labelled as “2”) a hydrophobic barrier between the two CYSABs. (c) galvanostatic discharge curves of battery packs 1 and 2. (d) digital watch powered by a bracelet containing two series-connected CYSABs.

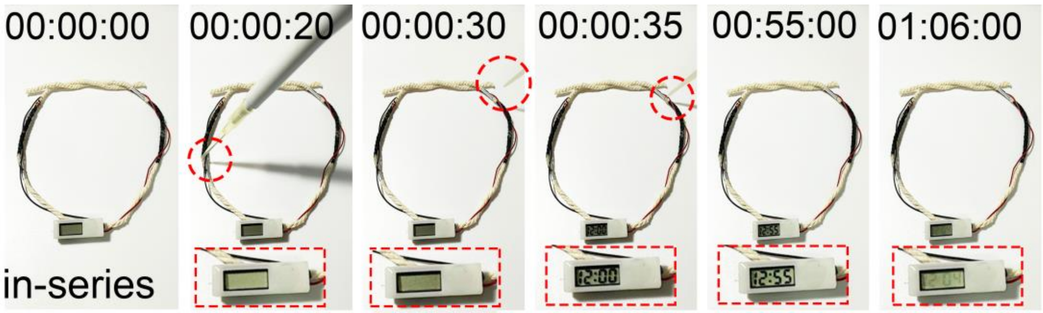

Figure S19. Series of images extracted from a video illustrating the working process of a digital watch powered by 2 in-series CYSABs after the addition of a 100 μL NaCl solution (100 mM) to each device.

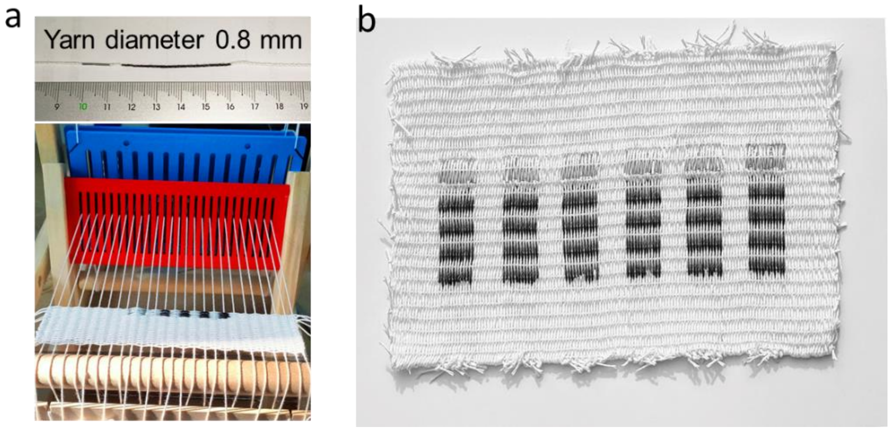

Figure S20. Weaving process of an energy fabric with the CYSABs as warp.

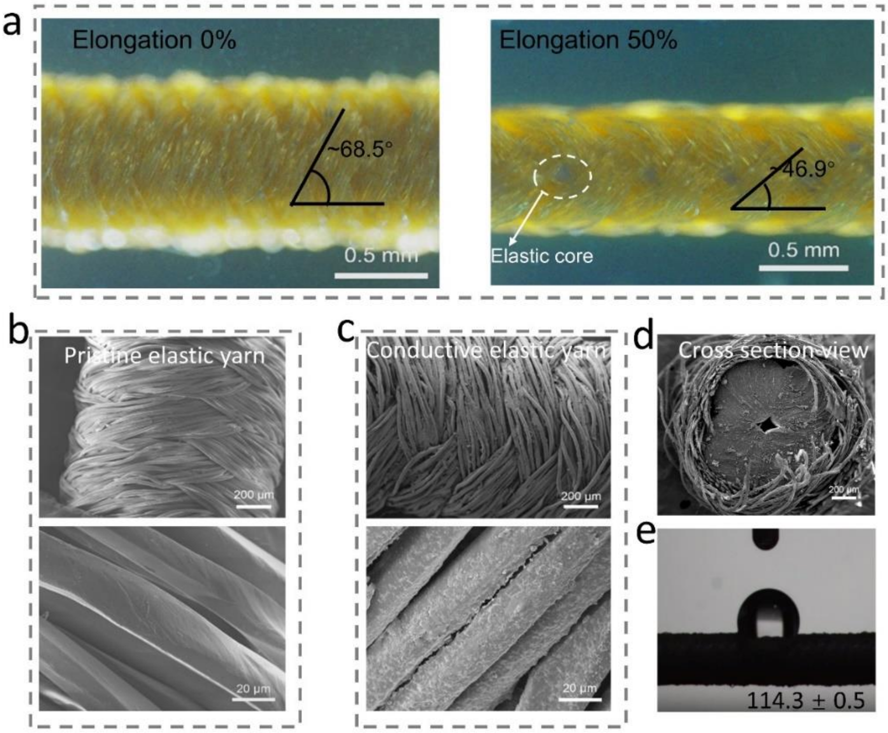

Figure S21. (a) Microscopic images of a pristine elastic yarn during various stretching states (elongations of 0% and 50%). SEM images of the (b) pristine elastic yarn and (c) PEDOT:PSS-coated elastic yarn. (d) Cross-section image of the conductive elastic yarn. (e) Contact angle image of the conductive elastic yarn.

A strain sensor was fabricated with a core-sheath-structured elastic yarn, which consists of a polyester fiber sheath and an elastic rubber core. The carbon black suspension was mixed with PEDOT:PSS at a ratio of 1:3 to prepare the conductive ink. The elastic yarn was soaked in the ink, followed by drying at 60 °C for 1 h. The resistance of the strain sensor could be adjusted by repeating the soaking-drying procedures.

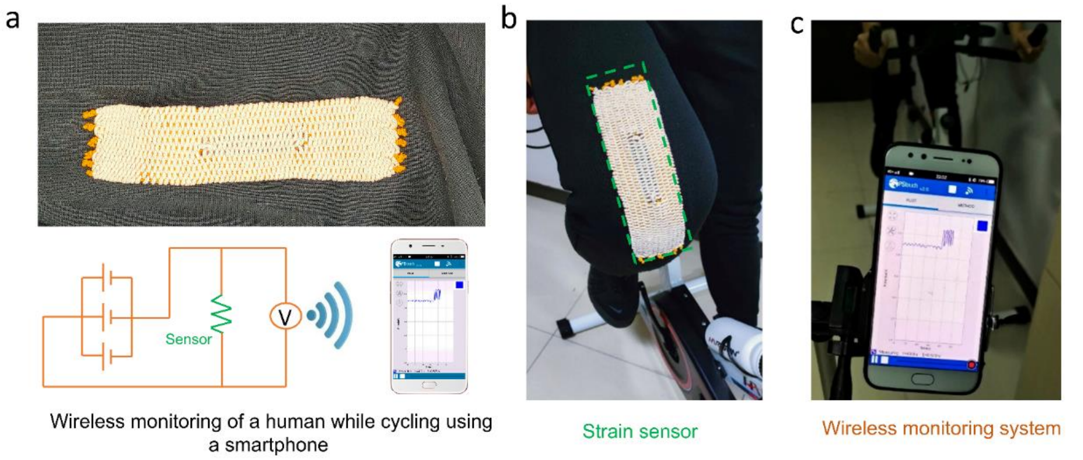

Figure S22. (a) Fabric-based strain sensor stitched onto black pants; (b) strain sensor at the stretching state during the on-body test; (c) wireless real-time monitoring of a human while cycling using a smart phone.

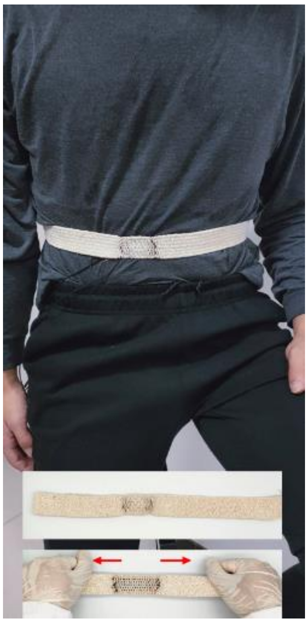

Figure S23. Attachment of a fabric-based strain sensor on a human abdomen for respiration monitoring. Inset: a fabric strain sensor in the resting (up) and stretching (bottom) states.

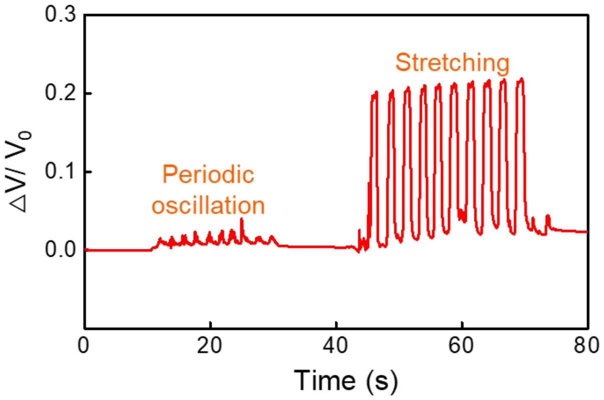

Figure S24. Effects of periodic oscillation on sensing performance of fabric-based self-powered sensing system.

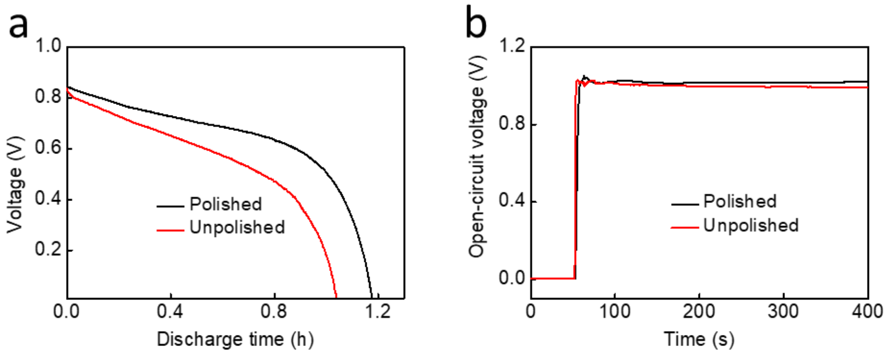

Figure S25. (a) Galvanostatic discharge curves of CYSABs fabricated with polished (black curve) and unpolished (red curve) Zn foils; (b) plots of the open-circuit voltage against time for the CYSABs fabricated with polished (black curve) and unpolished (red curve) Zn foils.

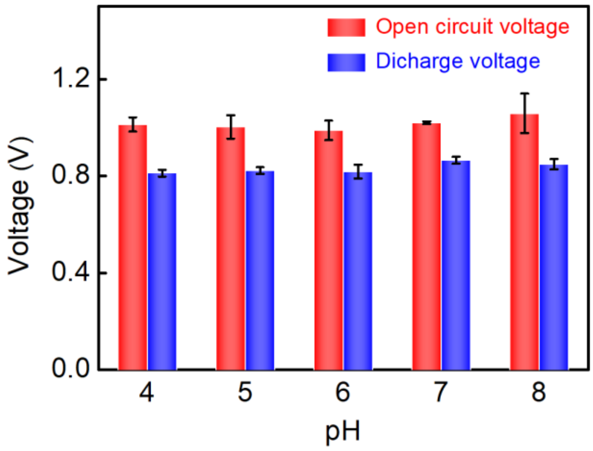

Figure S26. Effects of the NaCl solution pH on the open-circuit voltage and galvanostatic discharge voltage of the CYSABs. The data obtained from three independent experiments (n = 3) are presented as the mean ± standard deviations.

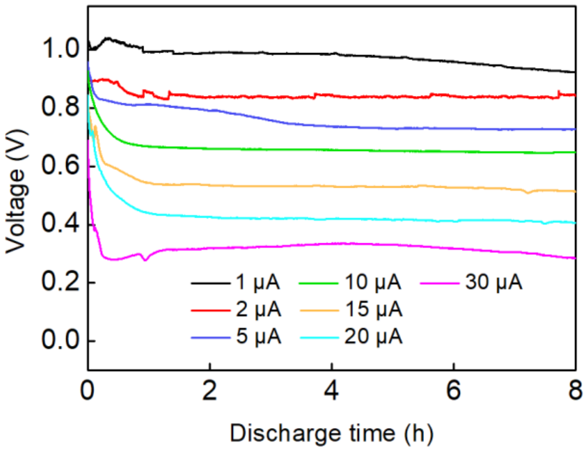

Figure S27. Effects of the current on the galvanostatic discharge behaviors of the CYSABs with continuous electrolyte supply.

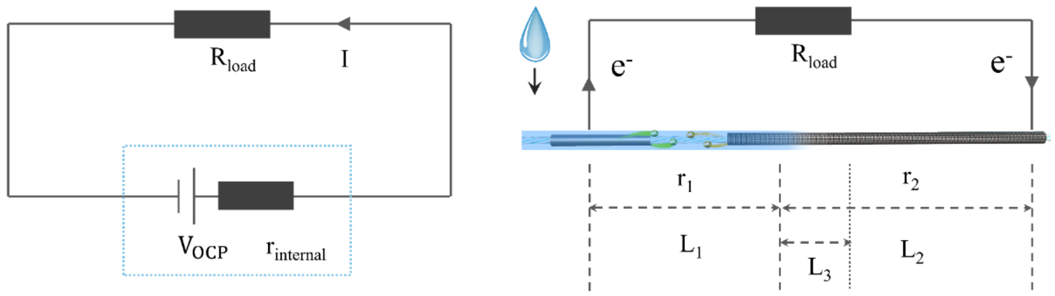

Figure S28. Schematic illustration of a working CYSAB to analyze the role of the carbon-black-coated fiber and salt bridge depending on their locations and the corresponding electric circuit.

Electron transfer between sweat and the carbon black nanoparticles

Increasing the length of the carbon-black-coated yarn immersed in sweat facilitated a more effective oxygen reduction catalytic area for the battery cathode. At the same time, a high concentration of salt ions, large oxygen reduction area, and large solid-liquid contact area facilitate electron transfer, which is one of the considerable advantages of using a longer carbon-black-coated thread as a battery cathode.

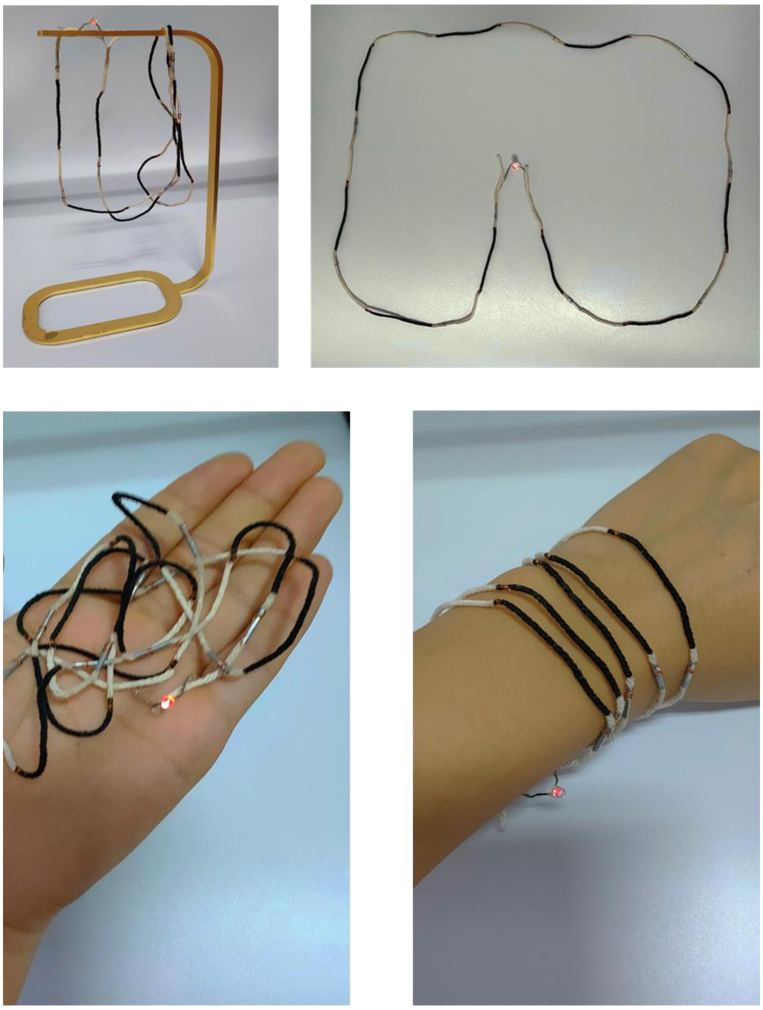

Figure S29. Photographs of a red LED powered by the curl/randomly coiled CYSABs.

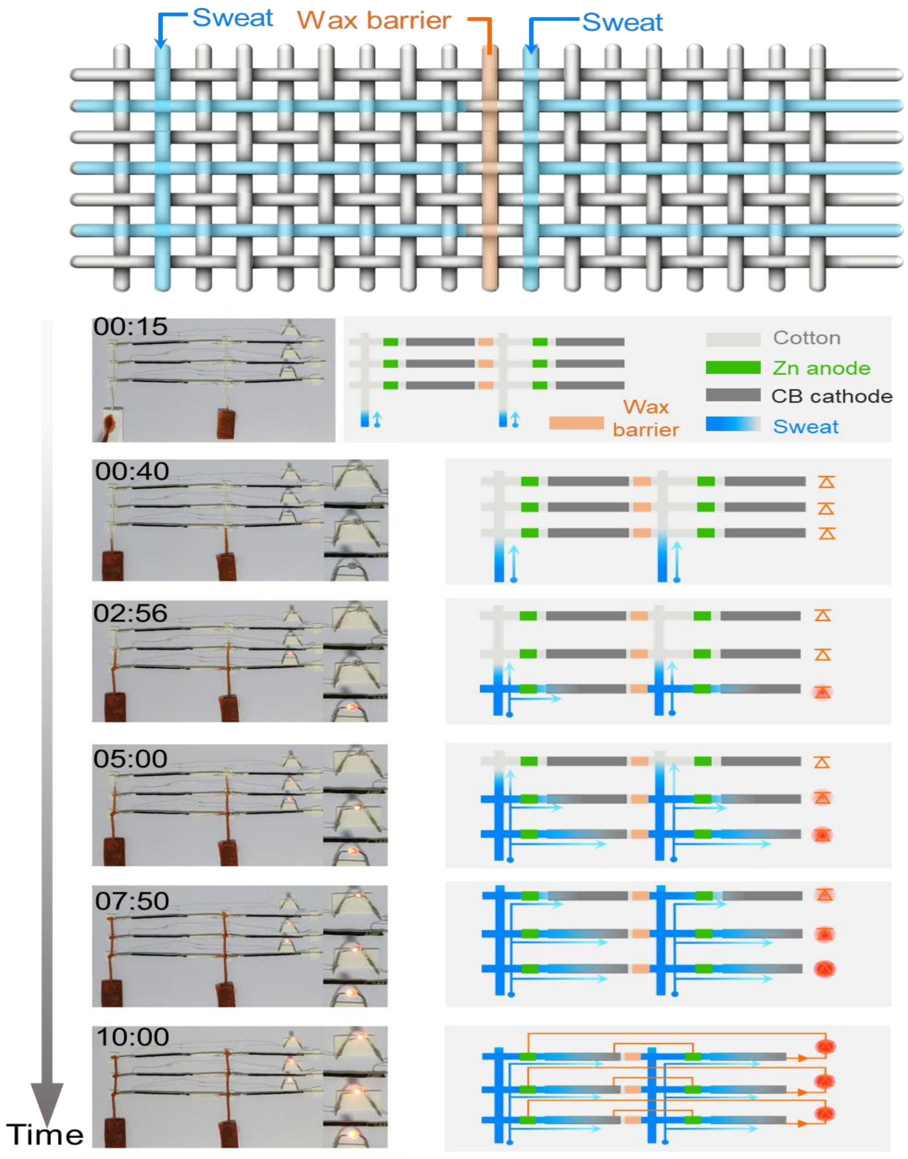

Figure S30. Time-dependent turning on of the LEDs due to the successive transport of liquid along the cotton yarns.

Table S1. Comparison of sweat-activated metal-based batteries.

Battery Category	Electrode Materials	Separator & Sweat Reservoir	Anode & Cathode Reactions	Maximum Output Voltage	Weavability	Washability (in water under stirring)	Reusability	Large-Scale Production	Device Type	Applications	Ref.
Zn-air	Zn foil - carbon black power	Cotton yarn	Anodic: $Zn - 2e^{-} \rightarrow Zn^{2+}$ Cathodic: $O_2 + 2H_2O + 4e^{-} \rightarrow 4OH^{-}$	~1.0 V	Yes	Yes	Yes	Yes	Yarn & Fabric	Power for an LED and a wearable strain sensor	**This work**
Zn-air	Zn foil - MWCNTs/ SWCNTs powder	Paper	Anodic: $Zn - 2e^{-} \rightarrow Zn^{2+}$ Cathodic: $O_2 + 2H_2O + 4e^{-} \rightarrow 4OH^{-}$	0.81 V	No	No	Not provided	No	Patch	Powering wireless heart-rate sensor/biosensing	1
Zn-Cu	Zn foil - Cu foil	Super-hygroscopic material	Anodic: $Zn - 2e^{-} \rightarrow Zn^{2+}$ Cathodic: $2H^{+} + 2e^{-} \rightarrow H_2$	0.57 V	Not provided	Not provided	Not provided	No	Patch	Powering a red LED	2
Mg-Ag/AgCl	Mg sheet - Ag/AgCl ink	Cellulose paper	Anodic: $Mg - 2e^{-} \rightarrow Mg^{2+}$ Cathodic: $2AgCl + 2e^{-} \rightarrow 2Ag + 2Cl^{-}$	~1.5 V	No	Not provided	No	No	Detachable electronic module	Wireless communication/heart rate sensor	3

Zn-air	Zn foil - PANI and CNT paper	Filter paper	Anodic: $Zn - 2e^{-} \rightarrow Zn^{2+}$ Cathodic: $O_2 + 2H_2O + 4e^{-} \rightarrow 4OH^{-}$	Not provided	No	No	Not provided	No	Patch	Charging for supercapacitors	4
Mg-Ag/AgCl	Mg foil-silver ink	Paper	Anodic: $Mg - 2e^{-} \rightarrow Mg^{2+}$ Cathodic: $2AgCl + 2e^{-} \rightarrow 2Ag + 2Cl^{-}$	~1.6 V	No	No	No	No	Patch	Sweat conductivity monitoring	5
Zn-Ag_2O	Zn flake inks-Ag_2O inks	Textile	Anodic: $Zn - 2e^{-} \rightarrow Zn^{2+}$ Cathodic: $Ag_2O + 2e^{-} \rightarrow 2Ag + O^{2-}$	~1.2 V	Not provided	Not provided	Not Mentioned	No	Patch	Powering a temperature sensor	6

[1] H. Wu, L. Xu, Y. Wang, T. Zhang, H. Zhang, C. R. Bowen, Z. L. Wang, Y. Yang, ACS Energy Letters 2020, 5, 3708.

[2] X. Zhang, J. Yang, R. Borayek, H. Qu, D. K. Nandakumar, Q. Zhang, J. Ding, S. C. Tan, Nano Energy 2020, 75, 104873.

[3] A. J. Bandodkar, S. P. Lee, I. Huang, W. Li, S. Wang, C. J. Su, W. J. Jeang, T. Hang, S. Mehta, N. Nyberg, P. Gutruf, J. Choi, J. Koo, J. T. Reeder, R. Tseng, R. Ghaffari, J. A. Rogers, Nature Electronics 2020, 3, 554.

[4] Z. Luo, Y. Wang, B. Kou, C. Liu, W. Zhang, L. Chen, Energy Storage Materials 2021, 38, 9.

[5] L. Ortega, A. Llorella, J. P. Esquivel, N. Sabaté, Microsystems & Nanoengineering 2019, 5, 3.

[6] J. Lv, G. Thangavel, Y. Li, J. Xiong, D. Gao, J. Ciou, M. W. M. Tan, I. Aziz, S. Chen, J. Chen, X. Zhou, W. C. Poh, P. S. Lee, Science Advances 2021, 7, eabg8433.
